# Targeting Dnmt3a/m5C/RelA Axis Attenuates Microglia Inflammatory Response and Improves Postoperative Recovery in Chronic Compressive Cervical Spinal Cord Injury

**DOI:** 10.1002/advs.75995

**Published:** 2026-06-09

**Authors:** Tianyu Qin, Yuan Jiang, Yongheng Xie, Huanwei Qu, Wenbin Yan, Jingle Chen, Yining Chen, Huayi Li, Naibo Feng, Jiajun Wu, Chao Zhang, Zhengqi Huang, Ming Shi, Zhihuai Deng, Guozhi Xiao, Houqing Long

**Affiliations:** ^1^ Division of Spine, Department of Orthopedics, Shenzhen People's Hospital (The Second Clinical Medical College, Jinan University, The First Affiliated Hospital Southern University of Science and Technology) Shenzhen China; ^2^ Shenzhen Key Laboratory of Musculoskeletal Tissue Reconstruction and Function Restoration Shenzhen China; ^3^ Division of Spine, Department of Orthopedics, Changde Hospital, Xiangya School of Medicine Central South University Changde China; ^4^ Department of Orthopedics, Tianjin Hospital Tianjin University Tianjin China; ^5^ Department of Trauma Orthopedics, Weifang People's Hospital Shandong Second Medical University Weifang China; ^6^ Department of Orthopedics, Sun Yat‐sen Memorial Hospital Sun Yat‐sen University Guangzhou China; ^7^ Department of Biochemistry Homeostatic Medicine Institute School of Medicine Guangdong Provincial Key Laboratory of Cell Microenvironment and Disease Research Southern University of Science and Technology Shenzhen China; ^8^ Guangdong Provincial Clinical Research Center for Geriatrics Shenzhen Clinical Research Center for Geriatrics Department of Geriatrics Shenzhen People's Hospital Shenzhen China

**Keywords:** chronic compressive spinal cord injury, neuroinflammation, microglia, Dnmt3a, m5C, gene therapy

## Abstract

Chronic compressive cervical spinal cord injury (cCSCI) is a major cause of adult spinal cord dysfunction. Surgical decompression is the primary treatment; however, microglia‐driven neuroinflammation often hampers postoperative recovery. This study was conducted to investigate the role of 5‐methylcytosine (m5C) in sustaining pro‐inflammatory microglial states following decompression. Using a mouse cCSCI model, we performed single‐nucleus RNA sequencing (snRNA‐seq) to map microglial states, calculated an m5C‐regulator activity score, and validated m5C changes via dot blot. We examined the Dnmt3a–RelA pathway in primary microglia through siRNA knockdown, m5C‐RIP, and mRNA stability assays. Therapeutic potential was assessed with intrathecal AAV‐shDnmt3a. SnRNA‐seq revealed a dominant pro‐inflammatory microglial subpopulation (C1) post‐decompression, enriched for NF‐κB signaling. The C1 subset exhibited an elevated m5C score and increased Dnmt3a expression. Dnmt3a knockdown reduced m5C enrichment on RelA transcripts, destabilized RelA mRNA, and suppressed NF‐κB activation. In vitro, Dnmt3a silencing reduced pro‐inflammatory marker expression and cytokine release, and microglia‐targeted AAV‐shDnmt3a improved neurological recovery in cCSCI mice. These findings underscore the potential of targeting the microglia Dnmt3a/m5C/RelA axis to enhance postoperative recovery.

## Graphical abstract

A pro‐inflammatory microglial subset persists after decompression in chronic compressive cervical spinal cord injury and shows enhanced Dnmt3a‐driven m5C signaling. By stabilizing RelA mRNA, this axis sustains NF‐κB activation and postoperative neuroinflammation. Microglia‐targeted AAV‐shDnmt3a suppresses inflammatory responses and improves functional recovery, highlighting an epitranscriptomic therapeutic avenue for postoperative repair.

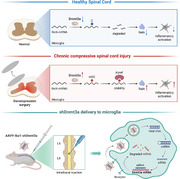

## 1 Introduction

Chronic compressive cervical spinal cord injury (cCSCI), which is also referred to as degenerative cervical myelopathy (DCM), is a progressive disorder characterized by spinal cord dysfunction [1]. Common etiological factors include cervical intervertebral disc herniation, ossification of the posterior longitudinal ligament, and hypertrophy of the ligamentum flavum [2]. These persistent compressive insults gradually lead to spinal cord damage, resulting in neurological deficits and a marked decline in patients’ quality of life [3]. Epidemiological studies have indicated that the incidence of cCSCI (12.33 per 1 000 person‐years) is substantially higher than that of traumatic spinal cord injury (TSCI; 0.13 per 1,000 person‐years) [4]. Unlike TSCI, which has an acute onset, cCSCI typically progresses insidiously and may lack characteristic symptoms in its early stages; thus, patients often present with established spinal cord dysfunction at the time of diagnosis [5, 6]. At this stage, even surgical decompression frequently fails to fully reverse the pre‐existing structural and functional pathological alterations [7, 8]. There is an urgent need to elucidate the molecular mechanisms underlying the limited neurological repair following cCSCI and develop effective therapeutic strategies that promote neurological functional recovery, given these clinical characteristics.

Prolonged mechanical compression of the spinal cord can trigger a cascade of pathological events that ultimately establishes a persistent neuroinflammatory microenvironment. Initially, reduced local perfusion leads to chronic ischemia and tissue hypoxia, which, in turn, induces endothelial dysfunction and disrupts the blood–spinal cord barrier (BSCB) [5]. Subsequently, increased vascular permeability permits the extravasation of neurotoxic substances and inflammation‐associated factors, further promoting microglial activation [9]. Under these conditions, microglia acquire a pro‐inflammatory activation state, characterized by increased iNOS and CD86 expression and the enhanced production of pro‐inflammatory cytokines, including TNFα, IL‐1β, and IL‐6. These inflammatory mediators exacerbate demyelination and axonal degeneration, induce oligodendrocyte apoptosis, and ultimately impair neural plasticity, thereby hindering functional recovery [10, 11, 12]. Although these processes have been widely observed, the upstream regulatory mechanisms by which microglia mediate neuroinflammation in cCSCI remain to be fully elucidated.

RNA methylation is an important epitranscriptomic mechanism that regulates gene expression. Among these modifications, 5‐methylcytosine (m5C) is a chemical modification widely distributed on mRNA and tRNA, influencing RNA stability, splicing, and translational efficiency [13]. The regulation of m5C modification relies on a dedicated enzymatic system comprising writers, readers, and erasers. Writers, such as the NSUN and DNMT families, catalyze cytosine methylation, readers (e.g., YBX1) recognize m5C marks and mediate downstream functions, while erasers (such as the TET family) remove m5C modifications [14, 15, 16]. Recent studies have shown that m5C participates in the development of neuronal apoptosis and inflammatory responses in TSCI by regulating the stability and expression levels of key transcripts [17, 18]. However, the role of m5C modification in cCSCI has not been systematically investigated, especially the mechanisms by which it regulates gene stability and modulates functional activity in microglia.

This study was conducted to test the hypothesis that aberrant m5C modification programs sustain pro‐inflammatory microglial states, thus limiting neurological recovery after decompression in cCSCI. We aimed to: (i) delineate the microglial landscape across sham, compression, and post‐decompression conditions and identify inflammation‐associated subclusters, (ii) construct m5C regulatory maps at the single‐cell and subcluster levels, prioritizing microglial subsets with the strongest m5C signatures, (iii) determine whether Dnmt3a is associated with m5C enrichment and regulates the stability of immune‐regulatory transcripts, and (iv) evaluate whether inhibition of Dnmt3a can attenuate neuroinflammation and enhance functional recovery after decompression. This study provides a mechanistic foundation for perioperative intervention in cCSCI by establishing a mechanistic link among cell type–specific epitranscriptomic regulation, microglial activation, and postoperative neurological outcomes.

## 2 Materials and Methods

### 2.1 Animals

C57BL/6 mice (10 weeks old) were obtained from the Guangdong Provincial Laboratory Animal Center. The mice were housed under specific pathogen‐free (SPF) conditions at a constant temperature (23 ± 2°C) and humidity (55%), with a 12‐h light/dark cycle. Food and water were provided ad libitum. Only male mice were used in this study in order to minimize animal use and maintain experimental consistency.

### 2.2 Establishment of the cCSCI Model and Treatment

The cCSCI mouse model was established in our laboratory, as previously described [19]. In brief, 10‐week‐old mice were anesthetized via intraperitoneal injection of sodium pentobarbital (50 mg/kg). The absence of pain reflexes was confirmed, and the mice were placed in the prone position. The dorsal cervical fur was shaved, and the surgical area was disinfected with povidone–iodine. A midline incision was made along the C3–C7 spinous processes, and the paraspinal muscles were sequentially dissected to expose the lamina. The C6 lamina and ligamentum flavum were removed to expose the dura using the C7 spinous process as a landmark. A sterilized polyurethane sheet (FL‐m‐c02, Fulam Technology, China) was trimmed and inserted into the epidural space between the C4–C6 lamina and dura. Upon hydration, the polyurethane expanded, establishing the chronic spinal cord compression model. The muscles and skin were sutured in layers. In the sham group, only the C6 lamina was removed, and polyurethane implantation was not performed. In the decompression group, the C5 lamina was removed through the original incision, and the polyurethane was extracted.

Mice were randomly assigned to receive 10 µL of either AAV9‐Iba1‐shDnmt3a (hereafter referred to as AAV‐shDnmt3a) or AAV9‐Iba1‐shNC (hereafter referred to as AAV‐shNC) in order to investigate the role of Dnmt3a in post‐decompression microglial activation. Both vectors (1.5 × 10^12 vg/mL) were synthesized by Hanbio Biotechnology (Shanghai, China). A 15 mL centrifuge tube was placed under the lower body to slightly elevate the injection site. After shaving, the viral solution was loaded into a 25 µL Hamilton syringe fitted with a 33G needle. The L5–L6 intervertebral space, located above the iliac crest line, was gently palpated with forceps, with the tail lifted to widen the interspace. The needle was vertically inserted, and a distinct ‘loss of resistance’ accompanied by tail flicking indicated penetration of the ligamentum flavum. The needle was stabilized, and the viral solution was slowly injected.

### 2.3 BMS

Locomotor function was evaluated using the Basso Mouse Scale (BMS; score range 0–9) [20]. Every mouse was assessed during an open‐field session, with scoring based on hindlimb joint movement, weight support, plantar stepping, fore–hindlimb coordination, paw placement, trunk stability, and tail position.

### 2.4 Inclined Plane Test

Hindlimb strength and balance were assessed using the inclined plane test. In this test, the mice were placed on an adjustable platform, and the inclination angle was gradually increased. The maximum angle at which mice could maintain their position for ≥ 5 s without sliding was recorded.

### 2.5 Forelimb Wire Hang Test

The forelimb hanging test was performed to assess neuromuscular grip strength. The mice were placed on a wire cage lid, which was gently inverted once they had grasped it. The latency to fall was recorded. The lid was positioned approximately 15 cm above a cushioned surface to minimize the risk of injury from falling.

### 2.6 Tissue Processing and Histopathology

At the end of the experiments, the mice were perfused transcardially with pre‐cooled PBS followed by 4% paraformaldehyde (PFA). The spinal cords were then harvested, post‐fixed in 4% PFA at 4°C overnight, and cryoprotected in 20% sucrose until they sank. The tissues were embedded in optimal cutting temperature (OCT) compound, snap‐frozen, and sectioned at 15 µm using a cryostat. For hematoxylin and eosin (H&E) staining, the sections were stained, dehydrated through graded ethanol, cleared in xylene, and mounted. For immunofluorescence, the sections were permeabilized with 0.3% Triton X‐100, blocked with 10% normal goat serum, and incubated with primary antibodies (see Table S4) overnight at 4°C. Subsequently, the sections were incubated with fluorophore‐conjugated secondary antibodies, then mounted in DAPI‐containing medium.

### 2.7 SnRNA‐Seq and Data Analysis

**FIGURE 1:**
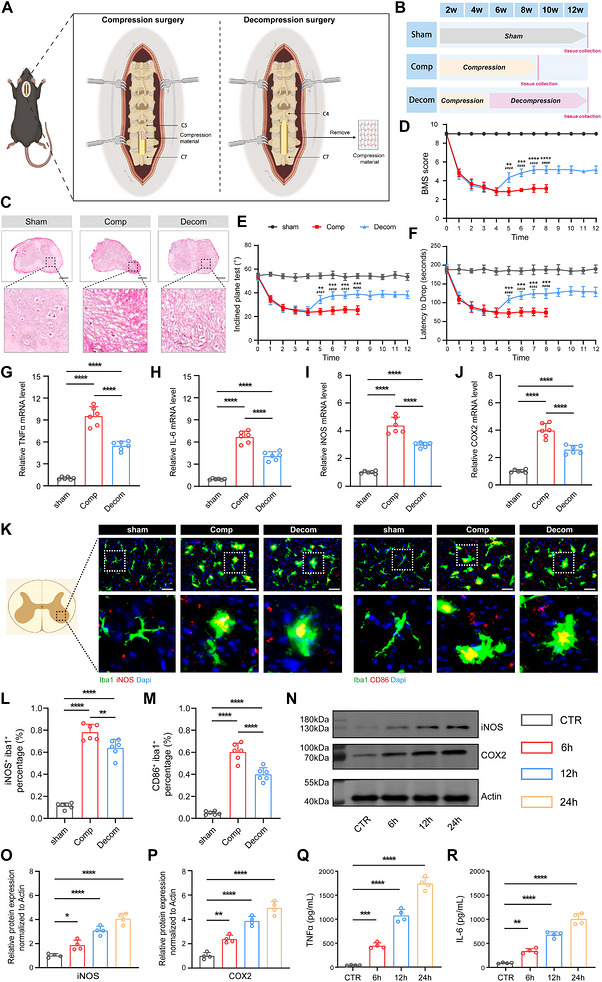
Microglia‐mediated neuroinflammation is associated with incomplete neurological recovery after decompression in cCSCI. (A) Schematic illustration of the establishment of the cCSCI mouse model and the subsequent decompression procedure. In the compression model, the C6 lamina and ligamentum flavum were removed to expose the dura, and a trimmed polyurethane sheet was inserted into the epidural space between the lamina and dura. For decompression, the C5 lamina was removed through the original incision, and the polyurethane sheet was extracted. (B) Experimental timeline showing grouping and sample collection points for Sham, Compression (Comp), and Decompression (Decom) mice. (C) Representative H&E staining of spinal cord sections showing reduced tissue area and cavitation after compression, with partial restoration following decompression. Scale bar = 500 µm. (D–F) Behavioral evaluation of motor function using the BMS, inclined‐plane test, and forelimb‐hanging test (n = 6/group). Symbols indicate comparisons: * vs Comp; # vs Sham. (G–J) qPCR analysis of pro‐inflammatory mediators TNFα, IL‐6, iNOS, and COX2 in spinal tissues. (K) Representative immunofluorescence images showing colocalization of iNOS or CD86 (red) with microglial marker Iba1 (green) in spinal cord sections. Scale bar = 30 µm. (L, M) Quantification of iNOS^+^Iba1^+^ and CD86^+^Iba1^+^ microglia demonstrating persistent pro‐inflammatory microglial activation after decompression. (N–P) Western blotting and densitometric analyses of iNOS and COX2 protein expression in primary microglia stimulated with LPS (100 ng/mL) for 6–24 h. (Q, R) ELISA showing time‐dependent increases in secreted TNFα and IL‐6 in primary microglia supernatants after LPS treatment. Data are presented as means ± SDs; *p < 0.05, **p < 0.01, ***p < 0.001, ****p < 0.0001.

Mice were divided into the Sham, compression (Comp), and decompression (Decom) groups. Dissection and subsequent processing were applied to the injured spinal cord segments derived from the Comp and Decom groups, alongside the matching uninjured sections obtained from the Sham group. The time points for tissue collection are shown in Figure 1B. Nuclei were isolated from spinal cord tissue on ice. Tissues were lysed at 4°C in pre‐chilled lysis buffer, and gently triturated to facilitate dissociation. The lysate was passed through a 30‐µm cell strainer, and the filtrate was washed once by centrifugation, then subjected to density‐gradient centrifugation to enrich for nuclei. Nuclear integrity was assessed microscopically. after washing. The resulting nuclear suspension was then loaded onto the 10× Genomics Chromium platform for downstream library preparation. FastQC was used for the preliminary quality assessment of the raw sequencing data. BCL files were converted to FASTQ and processed using the Cell Ranger count pipeline.

Raw sequencing data were aligned to the mouse reference genome using Cell Ranger (v7.2.0). Downstream analyses were conducted using Seurat (v4.1.1). Doublets were identified with DoubletFinder and removed. Batch effects were corrected using Harmony (RunHarmony) [21]. Data were normalized, clustered, and reduced in dimension in Seurat. Highly variable genes (n = 2000) were identified from the batch‐corrected matrix using FindVariableFeatures. PCA was performed using RunPCA, and the first 20 principal components were used for downstream analyses. Clustering was performed with FindClusters (resolution = 0.6), followed by uniform manifold approximation and projection (UMAP) visualization using RunUMAP. Cell types were annotated using the CellMarker 2.0 database together with canonical markers. Differentially expressed genes (DEGs) were identified using FindMarkers. Functional enrichment analyses (Gene Ontology (GO) and Kyoto Encyclopedia of Genes and Genomes (KEGG)) were performed using clusterProfiler [22]. Pseudotime trajectories were reconstructed using Monocle (v2.22.0) [23]. After dimensionality reduction and cell ordering, cells were projected onto trajectories to identify branch structures; cells within the same branch were considered to share similar states. Branch‐dependent genes were identified using branched expression analysis modeling (BEAM).

### 2.8 Cell Culture and Treatment

Primary microglial cells were isolated from the whole brains of C57BL/6 mice (postnatal days 1–2), as previously described [24]. In brief, brain tissue was dissociated using a Neural Tissue Dissociation Kit (130‐092‐628, Miltenyi Biotec, Germany). The resulting mixed glial cells were cultured for 10–14 days in complete DMEM supplemented with 0.5 ng/mL recombinant mouse M‐CSF (416‐ML, R&D Systems, USA). Microglial cells were then enriched by positive selection with CD11b‐conjugated magnetic beads (130‐093‐634, Miltenyi Biotec, Germany). The purified primary microglial cells were maintained in DMEM containing 10% fetal bovine serum (FBS) and 1% penicillin–streptomycin. Immunocytochemistry for the microglial marker Iba1 confirmed a purity greater than 95%.

Purified primary microglial cells were seeded into 6‐well plates for gene silencing experiments. After the cells had firmly adhered, they were transfected with siRNA targeting Dnmt3a or a negative control siRNA using Lipofectamine 3000 (L3000015, Invitrogen, USA). The siRNA sequences are listed in Table S5. The cells were transfected with the pcDNA3.1‐RelA plasmid (Hanbio Biotechnology, China) or the corresponding empty vector control, also using Lipofectamine 3000 for gene overexpression experiments.

### 2.9 Flow Cytometry

Flow cytometry with CD86 staining was used to assess microglial inflammatory activation after lipopolysaccharide (LPS) stimulation. The cells were treated with LPS (100 ng/mL), collected, washed twice with PBS, resuspended in fluorescence‐activated cell sorting (FACS) buffer, and incubated with anti‐CD86 (BD Biosciences, 558703) at 4°C in the dark. After staining, the cells were washed, resuspended in FACS buffer, and analyzed on a BD Biosciences flow cytometer. The data were analyzed using FlowJo.

### 2.10 Enzyme‐Linked Immunosorbent Assay (ELISA)

TNFα and IL‐6 levels in culture supernatants were measured by ELISA. The cells were plated in 6‐well plates and treated as indicated. The supernatants were then collected and centrifuged at 1,000 × *g* for 10 min at 4°C to remove debris. TNFα and IL‐6 concentrations were determined using mouse ELISA kits (TNFα: BMS607‐3; IL‐6: BMS603‐2; ThermoFisher Scientific, USA).

### 2.11 Western Blotting

Cells were lysed in RIPA buffer containing protease and phosphatase inhibitors. The lysates were incubated on ice for 30 min and centrifuged at 12,000 × *g* for 15 min at 4°C. Subsequently, the supernatants were collected, and protein concentrations were determined using a BCA assay (PC0020, Solarbio, China). Equal concentrations of protein were mixed with 5× SDS loading buffer, boiled at 95°C for 5 min, separated by SDS–PAGE, and transferred to PVDF membranes (IPVH00010, Millipore, USA). The membranes were then blocked with 5% BSA for 1 h, incubated with primary antibodies (Table S4) overnight at 4°C, and then incubated with HRP‐conjugated secondary antibodies for 1 h at room temperature. Signals were detected using ECL reagents and imaged with a chemiluminescence system (ChemiScope 6100 Touch, Clinx, China).

### 2.12 Immunofluorescence

Cells were seeded on coverslips in 24‐well plates and allowed to attach overnight. After treatment, the cells were washed with PBS, fixed with 4% PFA for 15 min at room temperature, and permeabilized with 0.3% Triton X‐100 in PBS for 10 min. Subsequently, the cells were blocked with 10% normal goat serum for 1 h and incubated with primary antibodies (Table S4) overnight at 4°C. After three PBS washes, the cells were incubated with fluorescent secondary antibodies for 1 h in the dark and counterstained with DAPI. Images were acquired using a fluorescence microscope (IX73PIF, Olympus, Japan).

### 2.13 Quantitative Real‐Time PCR (qPCR)

Total RNA was isolated from cells using TRIzol reagent. RNA concentration and purity were assessed using a NanoDrop 2000 spectrophotometer. cDNA was synthesized using a HiScript II Q RT Reagent Kit (R222, Vazyme, China). qPCR was performed using a General‐Purpose High Sensitivity Dye‐based qPCR Kit (Q711, Vazyme, China) with β‐actin as the internal control. Relative expression was calculated using the 2^−ΔΔCt^ method. The primer sequences are listed in Table S6.

### 2.14 m5C Dot Blot

Mice were randomly assigned to three groups: Sham, Comp, and Decom. Spinal cord microglia were isolated from each group, as previously described [25]. In brief, spinal cords were rapidly dissected and kept on ice. Then, the tissues were minced into 2‐mm pieces and enzymatically dissociated. Digestion was terminated with a buffer containing 2% FBS, followed by gentle trituration and filtration through a 40 µm cell strainer to obtain a single‐cell suspension. The cells were then enriched via isotonic density‐gradient separation, washed, and resuspended in staining buffer. The suspension was incubated with fluorophore‐conjugated anti‐mouse CD11b and CD45 antibodies, washed, filtered, and subjected to FACS. After debris exclusion based on FSC/SSC, doublet discrimination, and live‐cell gating, the CD45^low CD11b^+ population was collected as microglia. Total RNA was immediately extracted from the sorted cells, denatured, and spotted onto nylon membranes (Sigma–Aldrich). Following UV crosslinking, the membranes were stained with methylene blue as a loading control, then blocked and incubated with an anti‐m5C primary antibody (ab214727, Abcam) at 4°C overnight. Signals were visualized by ECL and imaged after incubation with an HRP‐conjugated secondary antibody. Dot intensities were quantified using ImageJ, and m5C signals were normalized to the total RNA signal from methylene blue staining. Overall changes in m5C levels were calculated relative to the Sham controls.

### 2.15 RNA Immunoprecipitation (RIP)

RIP assays were performed using a Magna RIP RNA‐Binding Protein Immunoprecipitation Kit (17‐700, Millipore, USA) according to the manufacturer's directions. In brief, cell lysates were incubated with magnetic beads conjugated with 5 µg of anti‐Dnmt3a, anti‐NSUN2, or anti‐NSUN6 antibody at 4°C overnight, with normal IgG used as a negative control. After washing and proteinase K digestion, the co‐immunoprecipitated RNA was purified by phenol–chloroform extraction, reverse‐transcribed into cDNA, and subjected to qPCR analysis. The enrichment of RelA mRNA was normalized to the input.

### 2.16 m5C‐MeRIP

Total RNA was isolated from cells using TRIzol reagent. mRNA was enriched using Dynabeads Oligo(dT)25 magnetic beads. m5C‐RIP was performed using an m5C‐RIP Kit (Bes5204‐1, BersinBio, China) with an anti‐m5C antibody or an IgG control, together with Protein A/G magnetic beads. Enriched mRNA was denatured at 65°C for 5 min and then incubated with antibodies and beads at 4°C for 2 h with rotation. Immunoprecipitated RNA was recovered with TRIzol and purified using an RNeasy Mini Kit (74104, QIAGEN, Germany). Purified RNA and 10% input were analyzed by qPCR to evaluate m5C enrichment on the RelA transcript.

### 2.17 RNA Stability Assay

Actinomycin D (S8964, Selleck, USA) was added to cell cultures at 5 µg/mL. The cells were collected at 0, 3, 6, and 9 h, and total RNA was extracted for qPCR to assess the half‐life of RelA mRNA. Relative expression at each time point was normalized to the 0 h baseline. Nonlinear regression was performed in GraphPad Prism to calculate mRNA half‐life.

### 2.18 RNA‐Seq and Data Analysis

Total RNA was isolated from primary microglia treated with LPS and transfected with siRNA‐Dnmt3a using TRIzol reagent. Poly(A)+ mRNA was purified using an NEBNext Poly(A) mRNA Magnetic Isolation Module (NEB, E7490). Libraries were prepared using an Illumina TruSeq mRNA Sample Preparation Kit (RS‐122‐2101) and sequenced on an Illumina NovaSeq 6000 platform (paired‐end). DEGs were defined as genes with |log2 fold change| > 1 and *p* < 0.05. GO and KEGG enrichment analyses were performed using clusterProfiler (v3.18.1) [22].

### 2.19 Statistical Analysis

Statistical analyses were performed using GraphPad Prism (version 9.5.0, GraphPad Software, USA). The significance threshold was set at *p* < 0.05. Data normality was assessed using the Shapiro–Wilk test. Student's *t* test was used to compare differences between two groups. One‐way ANOVA (with Tukey's post hoc test) or two‐way ANOVA (with Šidák's post hoc test) was applied for comparisons involving more than two groups. When datasets failed to conform to normality requirements, the Mann–Whitney or Kruskal–Wallis tests were implemented. Investigators performing behavioral assessment and image quantification were blinded to group allocation.

## 3 Results

### 3.1 Microglia‐Mediated Neuroinflammation Is Associated With Incomplete Neurological Recovery After Decompression in cCSCI

A chronic cervical spinal cord compression model was established in C57BL/6 mice, followed by surgical decompression (Figure 1A). Animals were assigned to the Sham, Comp, and Decom groups, as outlined in the experimental timeline (Figure 1B). Histological assessment by H&E staining showed a marked reduction in spinal cord area in the Comp group, with partial restoration after decompression. High‐magnification images further revealed tissue cavitation and extracellular matrix degradation in both Comp and Decom spinal cords (Figure 1C). Consistent with these structural changes, BMS scores progressively declined during compression and plateaued at week 4. After decompression, locomotor function improved gradually and peaked at postoperative week 3, then stabilized at a level that remained significantly below that of Sham controls (Figure 1D). The inclined plane and forelimb wire hang tests similarly indicated improved limb strength after decompression without full recovery to Sham levels (Figure 1E,F).

We quantified pro‐inflammatory mediators in spinal cord tissue in order to determine whether persistent inflammation accompanied this incomplete recovery. qPCR showed robust upregulation of TNFα, IL‐6, iNOS, and COX2 expression in the Comp group compared with that in the Sham group; decompression reduced these elevations but did not normalize them (Figure 1G–J). In line with these transcript changes, immunofluorescence co‐staining of Iba1 with pro‐inflammatory markers (iNOS and CD86) demonstrated significantly higher proportions of iNOS^+^Iba1^+^ and CD86^+^Iba1^+^ cells in Decom mice than in Sham mice (Figure 1K–M). Together, these data suggest that microglia‐associated pro‐inflammatory activation persists after decompression and is associated with incomplete neurological recovery.

In vitro, LPS (100 ng/mL) was used to induce a pro‐inflammatory phenotype in primary mouse microglia. iNOS and COX2 protein levels increased in a time‐dependent manner following LPS exposure (Figure 1N–P), accompanied by marked increases in TNFα and IL‐6 secretion (Figure 1Q,R). Flow cytometry further confirmed a progressive rise in CD86^+^ microglia after LPS stimulation (Figure S1A,B), supporting the robustness of this In vitro inflammatory model.

### 3.2 snRNA‐Seq Identifies a Pro‐Inflammatory C1 Microglial Subpopulation That Dominates After Decompression in cCSCI

**FIGURE 2:**
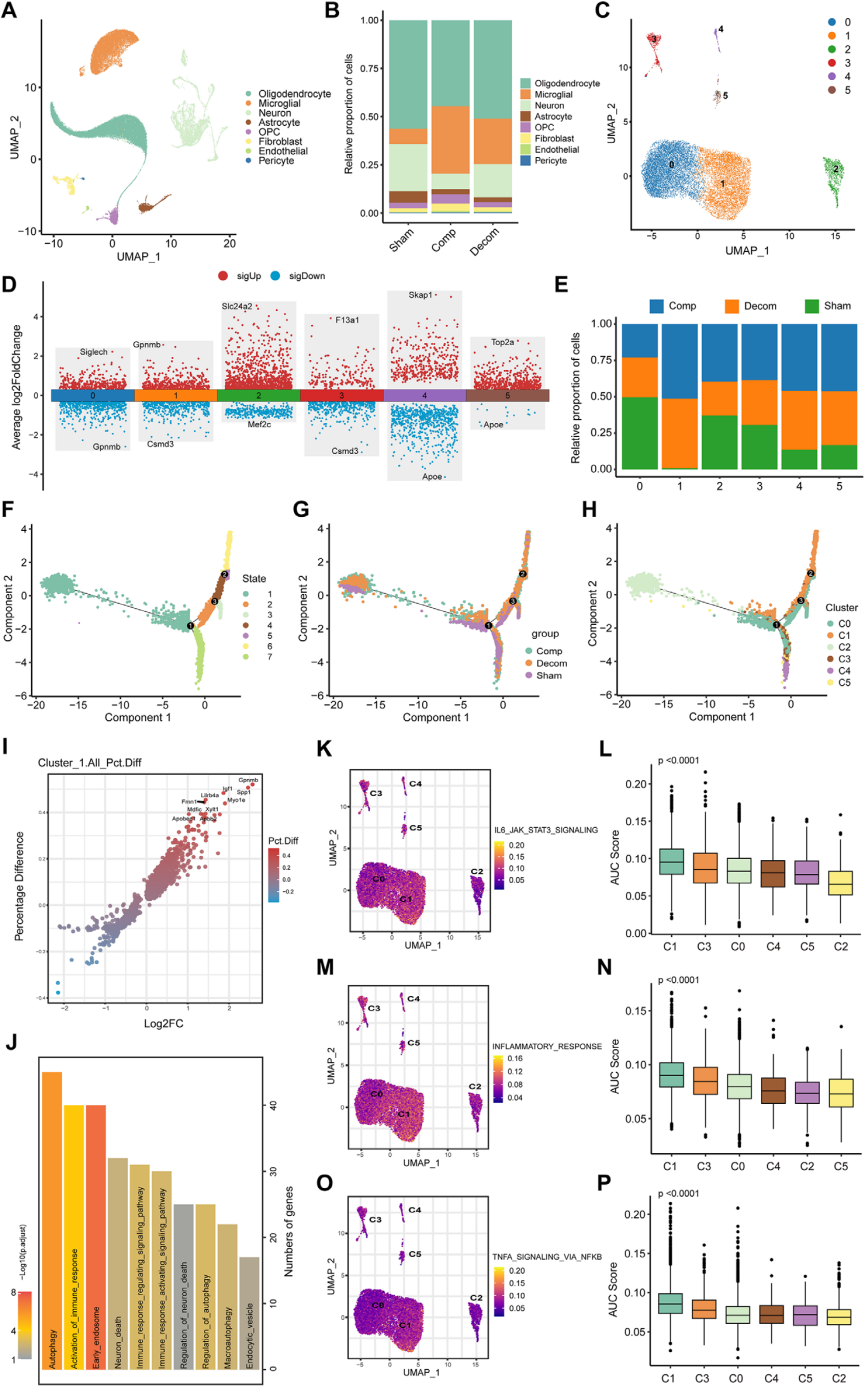
SnRNA‐seq identifies a pro‐inflammatory C1 microglial subpopulation dominating after decompression in cCSCI. (A) UMAP visualization of eight major spinal cord cell types identified by snRNA‐seq: oligodendrocytes, microglia, neurons, astrocytes, oligodendrocyte precursor cells (OPCs), fibroblasts, endothelial cells, and pericytes. (B) Relative proportions of cell types across the Sham, Compression (Comp), and Decompression (Decom) groups. (C) UMAP plot of six microglial subclusters (C0–C5) derived from the reclustering of 12,918 microglia. (D) Volcano plots showing representative differentially expressed genes (DEGs) among microglial subclusters, including Siglech, Gpnmb, Spp1, Cd63, and Apoe. (E) Distribution of microglial subcluster proportions across groups. (F–H) Pseudotime trajectory analysis of microglial differentiation using Monocle, with cells ordered by differentiation state (F), experimental group (G), and cluster identity (H). (I) Volcano plot showing C1‐specific DEGs compared with other subclusters. (J) GO analyses of C1 upregulated genes. (K–P) AUCell analysis showing elevated activity scores for IL6_JAK_STAT3_SIGNALING (K, L), INFLAMMATORY_RESPONSE (M, N), and TNFA_SIGNALING_VIA_NFKB (O, P) pathways in the C1 subcluster compared with other microglial populations. Data are presented as means ± SDs.

We performed snRNA‐seq on spinal cord tissues from Sham, Comp, and Decom mice to further characterize microglia‐associated neuroinflammation after decompression. Eight major cell types were identified: oligodendrocytes, microglia, neurons, astrocytes, oligodendrocyte precursor cells, fibroblasts, endothelial cells, and pericytes (Figure 2A and Figure S2A). The distribution of major cell types across groups is shown in Figure S2B, comprising 9,463 neurons and 12,918 microglia. Quantification revealed a pronounced expansion of microglia in the Comp group. Although microglial abundance decreased in the Decom group, it remained significantly higher than that in the Sham group (Figure 2B and Figure S2C).

Subclustering of 12,918 microglia resolved six subpopulations. Notably, although C1 microglia were rare in the Sham group (0.70%), they were the dominant subset in the Comp group (44.39%) and remained highly abundant in the Decom group (41.35%) (Figure 2C–E and Figure S2D,E). Trajectory inference further delineated seven transcriptional states across these subpopulations (Figure 2F and Figure S3A), with states 4–6 preferentially enriched in the Comp and Decom groups, largely driven by the C1 subset (Figure 2G,H and Figure S3B–Q). These results suggest that C1 microglia constitute a major inflammation‐associated population that persists after decompression.

We performed enrichment analyses using C1 DEGs (relative to other microglial subpopulations) in order to define the functional features of C1 microglia. GO terms were enriched for immune activation and immune response–activating pathways, and Hallmark analysis highlighted TNFA_SIGNALING_VIA_NFKB (Figure 2I,J and Figure S3R, S). AUCell scoring further supported heightened activity of IL6_JAK_STAT3_SIGNALING, INFLAMMATORY_RESPONSE, and TNFA_SIGNALING_VIA_NFKB programs in C1 microglia (Figure 2K–P). Collectively, these analyses indicate that C1 microglia exhibit a strongly pro‐inflammatory transcriptional profile.

### 3.3 The C1 Microglial Subpopulation Exhibits the Most Prominent m5C Regulatory Signature and Elevated Dnmt3a Expression After Decompression in cCSCI

**FIGURE 3:**
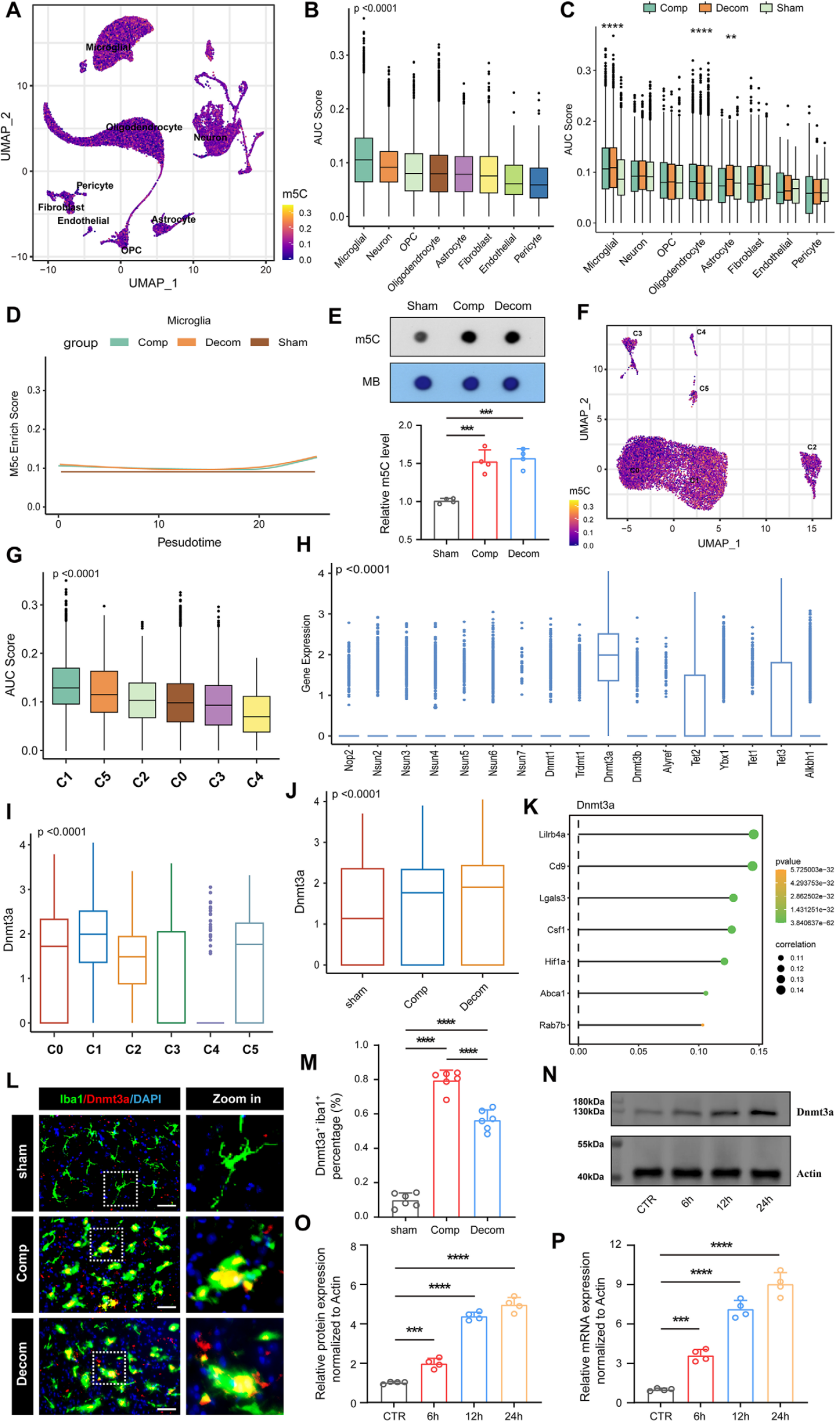
The C1 microglial subpopulation exhibits the most prominent m5C regulatory signature and elevated Dnmt3a expression after decompression in cCSCI. (A) UMAP visualization of all spinal cord cell types showing cell type specific m5C activity scores based on the m5C regulatory gene set. (B) Quantification of m5C scores across cell types. (C) Comparison of microglial m5C scores among groups. (D) Pseudotime trajectory of microglial m5C activity. (E) Dot blot analysis of RNA m5C levels in microglia from Sham, Comp, and Decom groups, with methylene blue (MB) staining as a loading control. (F, G) UMAP and AUCell analyses revealing that the C1 microglial subpopulation has the highest m5C regulatory gene set score among all subclusters (C0–C5). (H) Boxplots displaying expression levels of m5C regulators (Nsun2, Nsun3, Nsun4, Nsun5, Nsun6, Nsun7, Dnmt1, Dnmt3a, Dnmt3b, Trdmt1, Alkbh1, Tet1, Tet2, Tet3, and Apobec3). (I) Expression distribution of Dnmt3a across microglial subclusters (C0–C5). (J) Boxplot comparison of Dnmt3a expression across the Sham, Comp, and Decom groups. (K) Correlation analysis of Dnmt3a‐associated genes in the C1 subpopulation. (L) Representative immunofluorescence images showing colocalization of Dnmt3a (red) with microglial marker Iba1 (green) in spinal cord sections. Scale bar = 30 µm. (M) Quantification of Dnmt3a^+^ Iba1^+^ microglia proportions. (N) Western blotting of Dnmt3a expression in primary microglia stimulated with LPS for 6–24 h, with Actin as a loading control. (O, P) Densitometric and qPCR analyses showing time‐dependent upregulation of Dnmt3a at both the protein and mRNA levels following LPS stimulation. Data are presented as means ± SDs; *p < 0.05, **p < 0.01, ***p < 0.001, ****p < 0.0001.

We curated an m5C regulatory gene set from prior studies (Table S1) [15, 16] and calculated an AUCell‐based m5C machinery/signature score to explore whether m5C‐related epitranscriptomic regulation is associated with microglial inflammatory states in cCSCI. This score was significantly higher in microglia than in other major spinal cord cell types (Figure 3A,B). Moreover, microglia from Comp and Decom group mice displayed higher m5C machinery/signature scores than those from Sham group mice (Figure 3C). Along the inferred pseudotime trajectory, microglia from Comp and Decom group mice exhibited a progressive increase in the m5C machinery/signature score (Figure 3D and Figure S4A–G).

To validate m5C changes at the cellular level, microglia were isolated from spinal cords from Sham, Comp, and Decom group mice and subjected to m5C dot blot analysis. Global m5C levels were significantly higher in the microglia from the Comp and Decom groups than in those from Sham mice (Figure 3E). When the m5C machinery/signature score was compared across microglial subpopulations, C1 microglia exhibited the highest score (Figure 3F,G). Among m5C regulatory genes enriched in C1, Dnmt3a showed the strongest expression (Figure 3H), and Dnmt3a expression was consistently higher in C1 than in C0–C5 (Figure 3I). In addition, Dnmt3a expression in microglia was elevated in the Comp and Decom groups relative to that in the Sham group (Figure 3J and Figure S5A–P), which was further supported by immunofluorescence showing an increased proportion of Dnmt3a^+^ microglia in Comp and Decom mouse tissues (Figure 3L,M). In vitro, Dnmt3a expression also increased over time in primary microglia following LPS stimulation, as shown by western blotting and qPCR (Figure 3N–P).

Given the prominent inflammatory signaling in C1 microglia (Figure 2I–P and Figure S3R,S), we examined associations between Dnmt3a and genes within these enriched programs. Several genes, including Lilrb4a, Cd9, Lgals3, Csf1, Hif1a, Abca1, and Rab7b, showed positive correlations with Dnmt3a (*p* < 0.05; *r* > 0.1) and were highly expressed in C1 microglia (Figure 3K and Figure S6Q). Together, these findings suggest that Dnmt3a may be a prominent component of the m5C‐related program associated with the pro‐inflammatory C1 microglial state persisting after decompression.

### 3.4 Dnmt3a Knockdown Attenuates LPS‐Induced Pro‐Inflammatory Activation in Microglia

**FIGURE 4:**
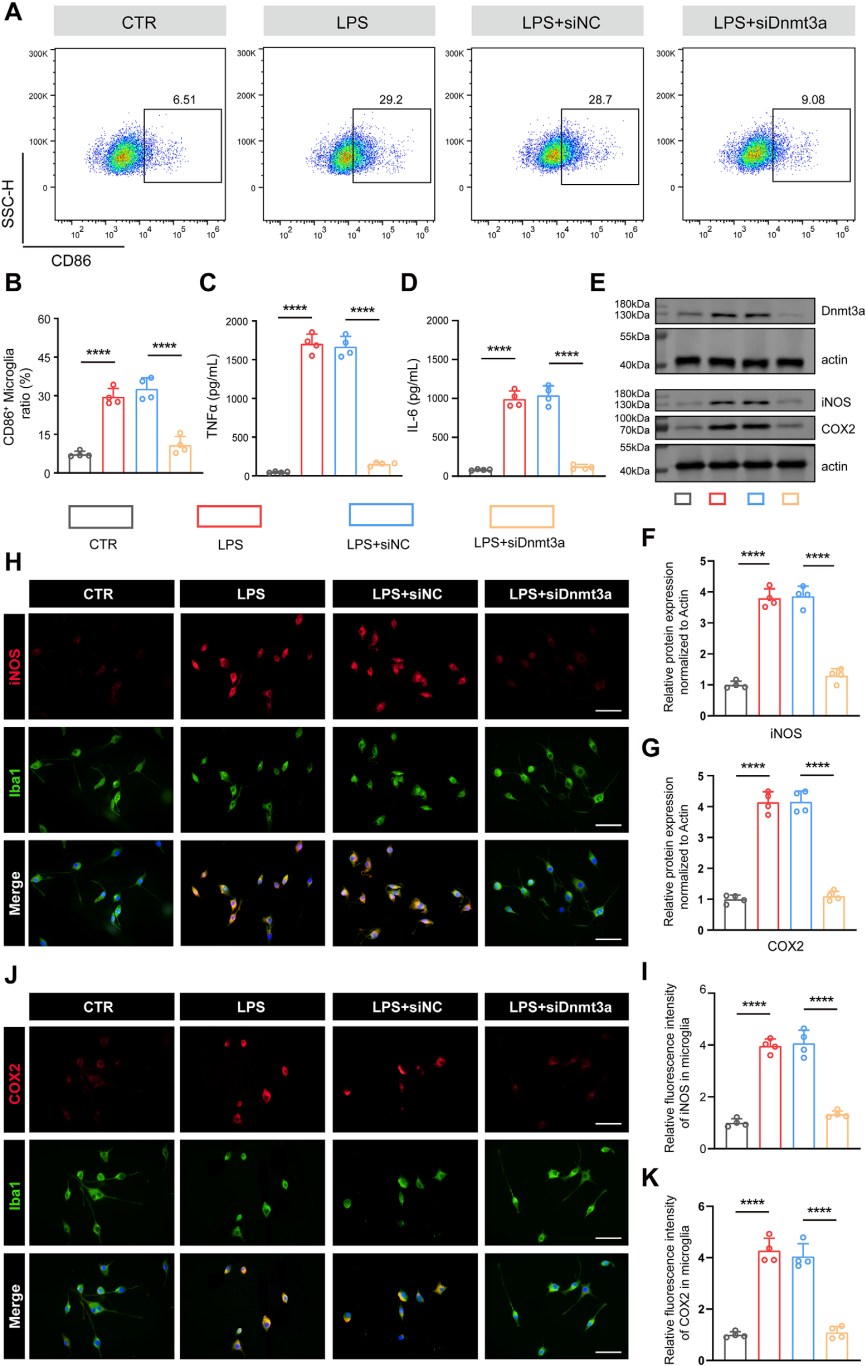
Dnmt3a knockdown attenuates LPS‐induced pro‐inflammatory activation in microglia. (A) Representative flow cytometry plots showing CD86^+^ microglial populations under different treatments: control (CTR), LPS, LPS + siNC, and LPS + siDnmt3a. (B) Quantification of CD86^+^ microglia percentages. (C–D) ELISA results showing that LPS stimulation increased TNFα and IL‐6 secretion, which were significantly decreased upon Dnmt3a knockdown. (E) Representative western blots showing expression of Dnmt3a, iNOS, and COX2 in primary microglia across groups, with actin as a loading control. (F–G) Densitometric quantification of iNOS and COX2 protein expression normalized to actin. (H–I) Representative immunofluorescence images of iNOS and COX2 in microglia. Scale bar = 50 µm. (J–K) Quantification of fluorescence intensity of iNOS and COX2 in microglia. Data are presented as means ± SDs. ****p < 0.0001.

Dnmt3a was silenced in primary microglia using siRNA (Figure S7A–D), followed by LPS stimulation (100 ng/mL) in order to test whether Dnmt3a contributes to microglial inflammatory activation. Flow cytometry showed that Dnmt3a knockdown significantly reduced the LPS‐induced increase in CD86^+^ microglia (Figure 4A,B). Consistently, ELISA demonstrated that LPS‐induced TNFα and IL‐6 secretion was markedly attenuated by Dnmt3a knockdown (Figure 4C,D).

At the protein level, LPS increased iNOS and COX2 expression by 3.8‐fold and 4.1‐fold, respectively, whereas Dnmt3a knockdown reduced iNOS and COX2 by 66.4% and 73.4% (Figure 4E–G). Immunofluorescence further showed parallel changes in iNOS and COX2 signal intensity, with LPS increasing fluorescence by ∼4.0‐fold and ∼4.3‐fold, and Dnmt3a knockdown reversing these increases (Figure 4H–K). These results suggest that Dnmt3a contributes to pro‐inflammatory microglial activation In vitro.

### 3.5 RelA Is a Candidate Transcript Showing Dnmt3a‐associated Changes in m5C Enrichment

**FIGURE 5:**
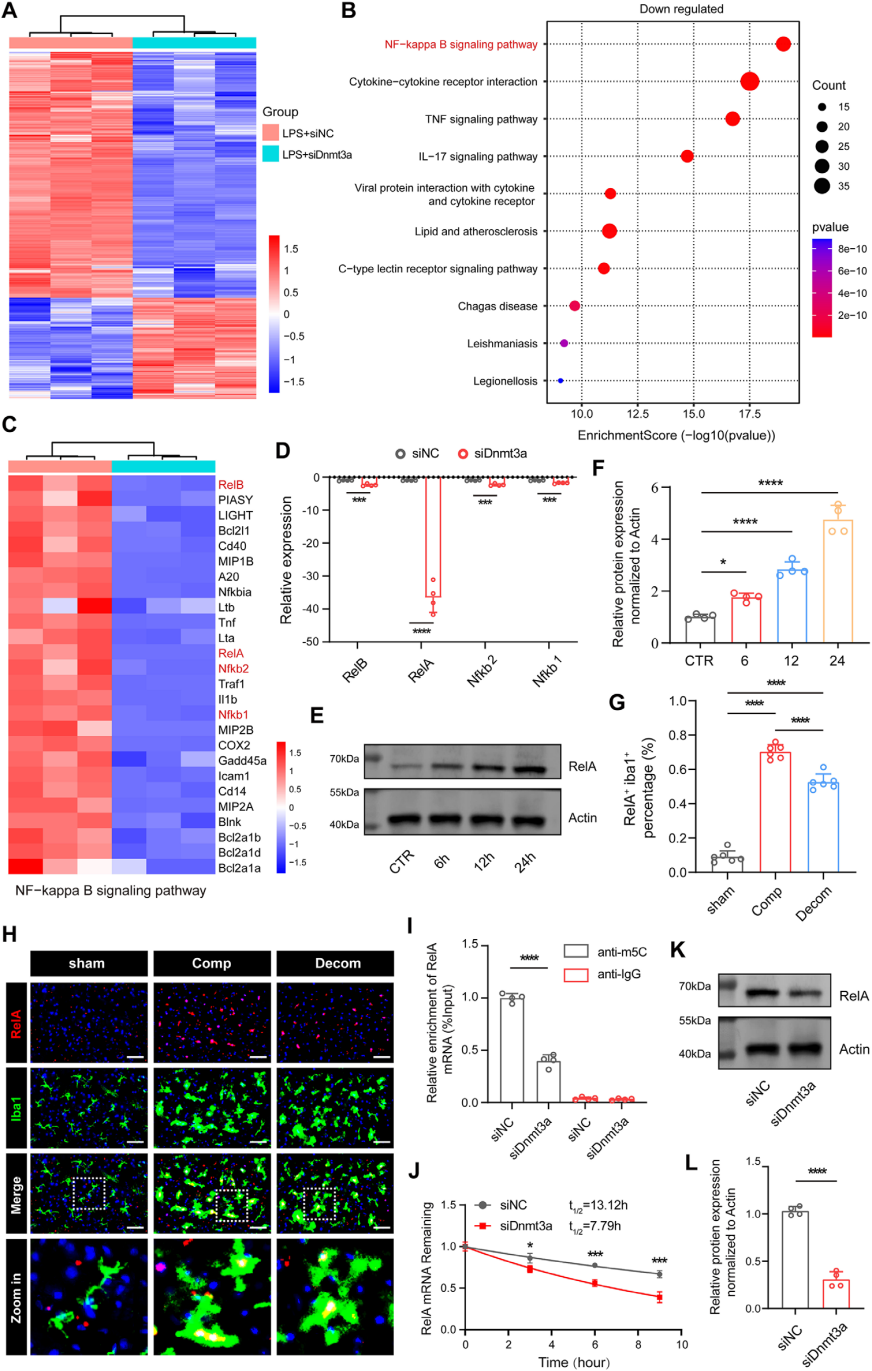
RelA is a candidate transcript showing Dnmt3a‐associated changes in m5C enrichment. (A) Heatmap showing the differential expression of genes in primary microglia transfected with siNC or siDnmt3a following LPS stimulation (n = 3/group). (B) KEGG pathway enrichment analysis of downregulated genes upon Dnmt3a knockdown, highlighting the NF‐κB signaling pathway as the most significantly affected pathway. (C) Heatmap of NF‐κB pathway–related genes. (D) qPCR validation of selected NF‐κB pathway genes (RelB, RelA, Nfkb2, and Nfkb1). (E–F) Western blot and quantification showing increased RelA protein expression after LPS treatment in primary microglia, peaking at 24 h. (G–H) Immunofluorescence staining of spinal cord sections showing colocalization of RelA (red) with the microglial marker Iba1 (green). Scale bar = 30 µm. (I) m5C‐RIP‐qPCR analysis showing significantly reduced m5C enrichment in RelA mRNA after Dnmt3a knockdown compared with siNC control. (J) Actinomycin D‐based RNA stability assay showing accelerated RelA mRNA decay in siDnmt3a‐transfected primary microglia. (K–L) Western blot and densitometric quantification confirming that Dnmt3a knockdown reduced RelA protein levels by approximately 70%. Data are presented as means ± SDs. ****p < 0.0001, ***p < 0.001, **p < 0.01, *p < 0.05.

We performed RNA‐seq under LPS stimulation and compared siDnmt3a‐ versus siNC‐transfected microglia to identify downstream targets of Dnmt3a in inflammatory microglia. In total, 515 DEGs were identified (Figure 5A and Figure S8A). The enrichment analysis of downregulated genes highlighted inflammation‐related processes (e.g., regulation of inflammatory response and positive regulation of cytokine production), and the NF‐κB signaling pathway was prominently implicated the KEGG analysis (Figure 5B and Figure S8B–D). Focusing on NF‐κB pathway transcription factors (Figure S9), RelA showed the largest change upon Dnmt3a knockdown (Figure 5C and Table S3), which was confirmed by qPCR (Figure 5D). RelA expression was also strongly induced by LPS in primary microglia (Figure 5E,F). In vivo, immunofluorescence revealed that RelA^+^ microglia numbers increased markedly in the Comp (70.4%) and Decom (52.7%) groups compared with those in the Sham group (9.1%), and RelA exhibited activation‐associated nuclear translocation in tissues from Comp and Decom mice (Figure 5G,H).

Because m5C can regulate transcript stability, we next assessed whether Dnmt3a influences RelA through m5C modification. Using m5C‐RIP, Dnmt3a inhibition was found to significantly reduce m5C enrichment on RelA transcripts (Figure 5I). Consistently, mRNA decay assays showed faster RelA mRNA degradation in siDnmt3a‐transfected microglia than in siNC control cells(Figure 5J). At the protein level, RelA was reduced by 70.1% following Dnmt3a knockdown (Figure 5K,L). Studies have shown that DNMT1 can recruit NSUN2 to mediate m5C methylation of mRNA, thereby stabilizing gene transcripts [26]. We next performed RIP‐qPCR for Dnmt3a, NSUN2, and NSUN6 in primary microglia to determine whether the effect of Dnmt3a on m5C modification of RelA mRNA is direct or indirectly mediated through other canonical m5C writers. Dnmt3a RIP showed significant enrichment of RelA mRNA compared with the IgG control, suggesting a direct association between Dnmt3a and the RelA transcript. In contrast, neither NSUN2 nor NSUN6 RIP showed appreciable enrichment of RelA mRNA under the same experimental conditions (Figure S10A). Together, these findings suggest that Dnmt3a is associated with increased m5C enrichment on RelA mRNA and reduced RelA mRNA decay.

We next examined two canonical m5C reader candidates, YBX1 and ALYREF to identify the m5C reader responsible for RelA transcript regulation. Three siRNAs targeting each reader were designed and synthesized for screening, and effective knockdown was used for subsequent validation experiments (Figure S10B–E). mRNA decay assays showed that YBX1 knockdown did not significantly affect the decay rate of RelA mRNA compared with that observed in siNC‐transfected cells (Figure S10F). Consistently, western blotting showed no obvious change in RelA protein expression after YBX1 silencing (Figure S10G, H). In contrast, ALYREF knockdown markedly accelerated RelA mRNA degradation and reduced RelA protein abundance (Figure S10I–K). Moreover, RIP‐qPCR demonstrated that ALYREF significantly enriched RelA mRNA (Figure S10L). These findings suggest that ALYREF, rather than YBX1, is the predominant functional reader mediating RelA mRNA stabilization in our experimental model.

### 3.6 RelA Partially Mediates the Pro‐inflammatory Effects of Dnmt3a in Microglia

**FIGURE 6:**
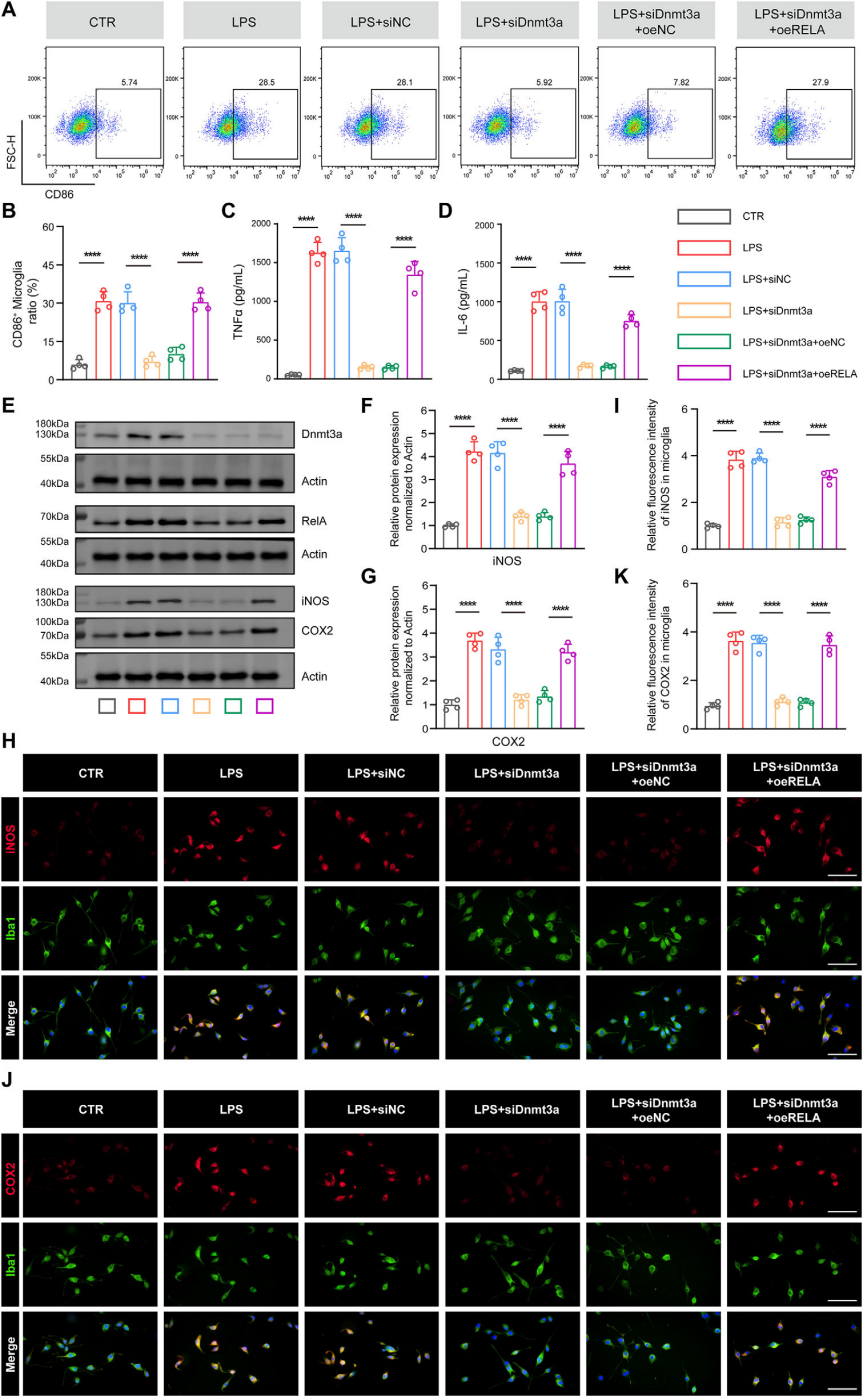
RelA partially mediates the pro‐inflammatory effects of Dnmt3a in microglia. (A) Flow cytometry analysis showing the percentages of CD86^+^ microglia under different treatment conditions: control (CTR), LPS stimulation, LPS + siNC, LPS + siDnmt3a, LPS + siDnmt3a + oeNC, and LPS + siDnmt3a + oeRELA. (B) Quantification of CD86^+^ microglia ratios. (C, D) ELISA analysis of TNFα and IL‐6 secretion in primary microglial supernatants. (E–G) Western blot and densitometric quantification of Dnmt3a, RelA, iNOS, and COX2 expression. (H–I) Quantification of fluorescence intensity for iNOS and COX2 in microglia, demonstrating that RelA overexpression reverses the inhibitory effect of Dnmt3a silencing on iNOS and COX2 expression. (J, K) Representative immunofluorescence images of iNOS and COX2 in primary microglia. Scale bar = 50 µm. Data are presented as means ± SDs. ****p < 0.0001, ***p < 0.001, **p < 0.01, *p < 0.05.

Next, we constructed a RelA overexpression plasmid (Figure S11A,B) and performed rescue experiments under LPS stimulation to test if RelA mediates the pro‐inflammatory effects of Dnmt3a. Dnmt3a knockdown reduced CD86^+^ microglia to 7.1%, whereas RelA overexpression restored CD86^+^ cells to 30.4% (Figure 6A,B). In parallel, RelA overexpression reversed the suppression of LPS‐induced TNFα and IL‐6 secretion caused by Dnmt3a knockdown (Figure 6C,D).

At the protein level, the reductions in iNOS and COX2 following Dnmt3a silencing were largely rescued by RelA overexpression (Figure 6E–G). Immunofluorescence showed consistent patterns, with Dnmt3a knockdown decreasing iNOS and COX2 by 70.5% and 68.6%, respectively, and RelA overexpression significantly restoring their expression (Figure 6H–K). Together with the m5C‐RIP and RNA stability data, these rescue experiments support RelA as a major downstream mediator through which Dnmt3a contributes to microglial inflammatory activation.

### 3.7 Targeted Inhibition of Microglial Dnmt3a Attenuates Neuroinflammation and Promotes Functional Recovery After Decompression in cCSCI

**FIGURE 7:**
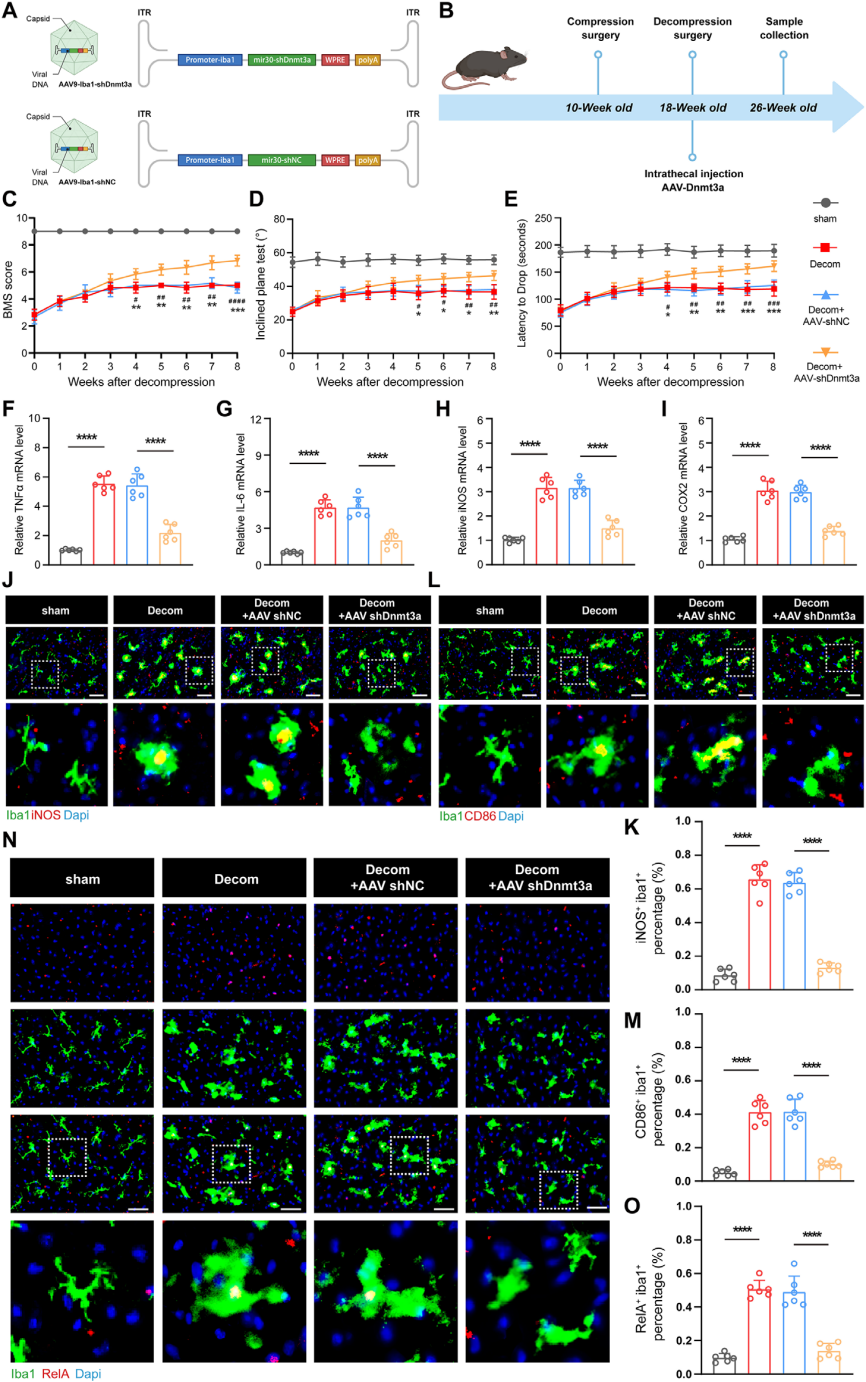
Targeted inhibition of microglial Dnmt3a attenuates neuroinflammation and promotes functional recovery after decompression in cCSCI. (A) Schematic representation of AAV9 constructs used for microglia‐targeted AAV‐shDnmt3a and the negative control (AAV‐shNC) under the Iba1 promoter. (B) Experimental timeline showing the establishment of the chronic compression model, decompression surgery, intrathecal AAV administration, and tissue analyses. (C–E) Behavioral assessments of motor function recovery following decompression, including BMS score, inclined plane test, and forelimb wire hanging test (n = 6/group). (F–I) qPCR analysis of pro‐inflammatory mediators (TNFα, IL‐6, iNOS, and COX2) in spinal cord tissues. Symbols indicate comparisons: * vs Decom; # vs. AAV‐shNC. (J) Representative immunofluorescence images showing co‐staining of iNOS (red) and Iba1 (green) in spinal cord sections from each group. Scale bar = 30 µm. (K) Representative immunofluorescence staining of CD86 (red) and Iba1 (green) in spinal cord sections. Scale bar = 30 µm. (L) Quantification of iNOS^+^ Iba1^+^ microglia. (M) Quantification of CD86^+^Iba1^+^ microglia. (N) Representative immunofluorescence images showing RelA (red) and Iba1 (green) staining. Scale bar = 30 µm. (O) Quantification of RelA^+^ Iba1^+^ microglia confirming reduced RelA expression following Dnmt3a knockdown. Data are presented as means ± SDs. ****p < 0.0001, ***p < 0.001, **p < 0.01, *p < 0.05.

For In vivo validation, we generated AAV vectors to knock down Dnmt3a in microglia (AAV‐shDnmt3a), with AAV‐shNC as the negative control (Figure 7A). To assess tolerability, we first monitored motor performance and body weight after AAV administration. No significant differences were observed among AAV‐shNC, AAV‐shDnmt3a, and control mice in BMS scores, inclined plane performance, or forelimb wire hang test outcomes over an 8‐week period (Figure S12A–C). Body weight increased steadily in all of the groups, indicating good tolerability under the experimental conditions (Figure S12D). We then performed immunofluorescence co‐staining of Dnmt3a with NeuN, GFAP, and Olig2 to further evaluate the cell type specificity of the AAV intervention. The results showed that AAV‐shDnmt3a did not induce a noticeable reduction in Dnmt3a signal in NeuN‐positive neurons, GFAP‐positive astrocytes, or Olig2‐positive oligodendroglial cells. In contrast, the decrease in Dnmt3a expression was primarily observed in Iba1‐positive microglia. (Figure S12E–L)

We then administered intrathecal AAV during decompression surgery in the cCSCI model and randomized animals into four groups: Sham, Decom, AAV‐shNC, and AAV‐shDnmt3a (Figure 7B). BMS scores in Decom mice plateaued by week 3, whereas AAV‐shDnmt3a‐treated mice continued to improve beyond week 3 and reached the highest functional level by week 8 (Figure 7C). Consistently, the inclined plane test and forelimb wire hang test demonstrated significantly improved limb strength in the AAV‐shDnmt3a group compared with AAV‐shNC controls (Figure 7D,E).

At the molecular level, qPCR showed that TNFα, IL‐6, iNOS, and COX2 were significantly reduced in AAV‐shDnmt3a spinal cords relative to AAV‐shNC (Figure 7F–I). Immunofluorescence further demonstrated a marked reduction in pro‐inflammatory microglia: iNOS^+^Iba1^+^ and CD86^+^Iba1^+^ cells decreased from 65.8% and 41.3% in Decom mice to 13.2% and 8.2% in AAV‐shDnmt3a‐treated mice, respectively (Figure 7J–M). In addition, AAV‐mediated Dnmt3a knockdown reduced the proportion of RelA^+^Iba1^+^ microglia and suppressed the activation‐associated nuclear translocation of RelA (Figure 7N,O), while effectively decreasing microglial Dnmt3a expression (Figure S12E, F). Collectively, these findings suggest that microglia‐targeted Dnmt3a inhibition attenuates post‐decompression neuroinflammation and improves neurological recovery in cCSCI mice.

## 4 Discussion

Although surgical decompression can arrest disease progression in cCSCI, a considerable proportion of patients do not achieve satisfactory functional recovery. In multicenter prospective studies, nearly half of patients do not reach the minimal clinically important difference (MCID) by 12 months postoperatively and retain significant residual functional deficits [27, 28]. These findings align with histopathological evidence showing that necrotic foci and cavitation‐like lesions frequently persist after decompression, underscoring that surgical decompression does not equate to histological restoration [29]. In light of our data, these clinical observations can be explained by a sustained state of neuroinflammation: in a mouse model of cCSCI, 8 weeks after decompression, levels of pro‐inflammatory cytokines such as TNFα and IL‐6 in spinal tissue remained markedly elevated compared with those in the controls; concurrently, the proportion of microglia exhibiting a pro‐inflammatory marker (iNOS and CD86) remained high. Further, paired cerebrospinal fluid analyses before and 3 months after surgery revealed that BSCB permeability recovers only partially after surgery and remains relatively elevated [30], suggesting that the persistent extravasation of blood‐derived molecules can repeatedly activate microglia and perpetuate this maladaptive inflammatory state.

Notably, most previous studies have treated microglia as a relatively homogeneous population, obscuring the question of “who drives inflammation” [31, 32, 33]. In this study, single‐nucleus transcriptomic analysis identified a pro‐inflammatory microglial subpopulation (C1). This subset remains dominant after decompression and is enriched for pro‐inflammatory pathways, including TNFA_SIGNALING_VIA_NFKB and IL6_JAK_STAT3_SIGNALING. Further analyses revealed that activity scores for m5C regulatory factors were most pronounced in the C1 subpopulation. Moreover, the m5C‐related regulator Dnmt3a was highly expressed in C1, suggesting that the pro‐inflammatory C1 microglial state is associated with enhanced Dnmt3a‐related epitranscriptomic regulation. Functionally, we observed that Dnmt3a knockdown markedly suppresses microglial inflammatory activation. In addition, it has been reported that the m5C eraser TET1 is upregulated in primary microglia in a mouse cerebral ischemia–reperfusion model and promotes M1 polarization through m5C‐mediated mechanisms [34], providing further support for a potential link between m5C regulation and microglia‐driven neuroinflammation in cCSCI.

The DNMT family has been traditionally regarded as a group of DNA methyltransferases. However, increasing evidence suggests that DNMT‐family proteins may also participate in RNA methylation‐related regulation [35, 36, 37]. In our study, inhibition of Dnmt3a in microglia reduces m5C enrichment on RelA transcripts and accelerates RelA mRNA decay. Notably, one recent study demonstrated that Dnmt1 can recruit NSUN2 to induce the m5C modification of target transcripts and enhance their stability [26], suggesting that DNMT‐family proteins may regulate RNA m5C either directly or indirectly through cooperation with canonical m5C writers. To further examine this effect in our model, we prioritized NSUN2 and NSUN6 for RIP‐qPCR validation, as these proteins are among the most well‐supported canonical candidates for mRNA m5C modification in the current literature [38, 39]. Under our experimental conditions, neither NSUN2 nor NSUN6 showed appreciable enrichment of RelA mRNA, whereas Dnmt3a exhibited clear binding to the RelA transcript. These findings indicate that Dnmt3a is the principal RelA‐associated regulatory factor in our model and that NSUN2/NSUN6 are not major direct RelA‐binding regulators. Importantly, our current evidence is derived from MeRIP rather than RNA bisulfite sequencing; thus, we could not identify the exact modified cytosines on RelA. Accordingly, the results should be interpreted as evidence at the transcript level rather than a site‐resolved mechanistic analysis.

RelA, the effector subunit of the canonical NF‐κB pathway, is typically retained in the cytoplasm as a p50/RelA heterodimer. Upon inflammatory stimulation, RelA translocates to the nucleus and induces the transcription of pro‐inflammatory genes, such as TNFα, IL‐1β, and iNOS [40]. Substantial evidence links RelA activation to microglial activation and inflammatory gene programs [41, 42, 43]. In our study, RelA was highly expressed in microglia in both the murine cCSCI model and the post‐decompression model, accompanied by enhanced nuclear translocation. Notably, AAV‐mediated Dnmt3a knockdown reversed these changes, thereby dampening microglia activation and promoting neurological functional recovery. Studies have shown that autophagy‐ and apoptosis‐related pathways can interact with inflammatory signaling and may contribute to the pathological microenvironment [44, 45]. However, these pathways were not directly examined in the present study and cannot be fully excluded. Importantly, m5C‐RIP, mRNA stability assay, and RelA rescue experiment data collectively support a model in which Dnmt3a promotes microglial inflammatory activation, at least in part, through m5C‐associated stabilization of RelA mRNA.

In clinical practice, many patients with cCSCI present with significant, and even irreversible, neurological damage at the time of diagnosis, thereby reducing the benefits of surgery [46]. Moreover, no pharmacological therapy has been demonstrated to enhance neurological recovery after decompression [47]. AAV‐based gene therapy may offer a potential strategy to help address this unmet need [48]. In the central nervous system (CNS), AAV vectors have shown comparatively favorable properties in preclinical and clinical studies, including relatively sustained transgene expression and the potential for cell‐ or region‐specific delivery through the combined selection of capsid and promoter [49, 50]. Guided by this rationale, we engineered an Iba1 promoter–driven AAV‐shDnmt3a to selectively inhibit microglial Dnmt3a at the time of decompression. This intervention markedly reduced the expression of pro‐inflammatory mediators in spinal cord tissue, decreased the proportion of pro‐inflammatory microglia, and enabled functional recovery to surpass the postoperative week‐3 plateau, resulting in improved behavioral outcomes at week 8. Although Iba1 is widely used for histological visualization of microglia in the CNS, it is not absolutely specific and may also label infiltrating macrophages under pathological conditions. Thus, the Iba1‐based In vivo readouts in the present study should be interpreted as predominantly microglial rather than exclusively microglial. Nevertheless, because infiltrating macrophages represented only a very small proportion of spinal cord cells in our cCSCI model [19], we consider that the Iba1‐positive population in this setting predominantly comprised microglia.

This study has several limitations. First, due to ethical and practical constraints, direct validation in human spinal cord tissue is not feasible; thus, the translational relevance of the Dnmt3a/m5C/RelA axis will require further evaluation in clinically accessible human samples or other human‐relevant systems in the future. Second, although intrathecal administration of AAV‐shDnmt3a was well tolerated during the observation period and did not produce obvious adverse effects on body weight or motor function, a systematic long‐term safety assessment has not yet been performed. Accordingly, extended follow‐up and more comprehensive biosafety evaluation will be necessary before considering clinical translation.

## 5 Conclusion

**FIGURE 8:**
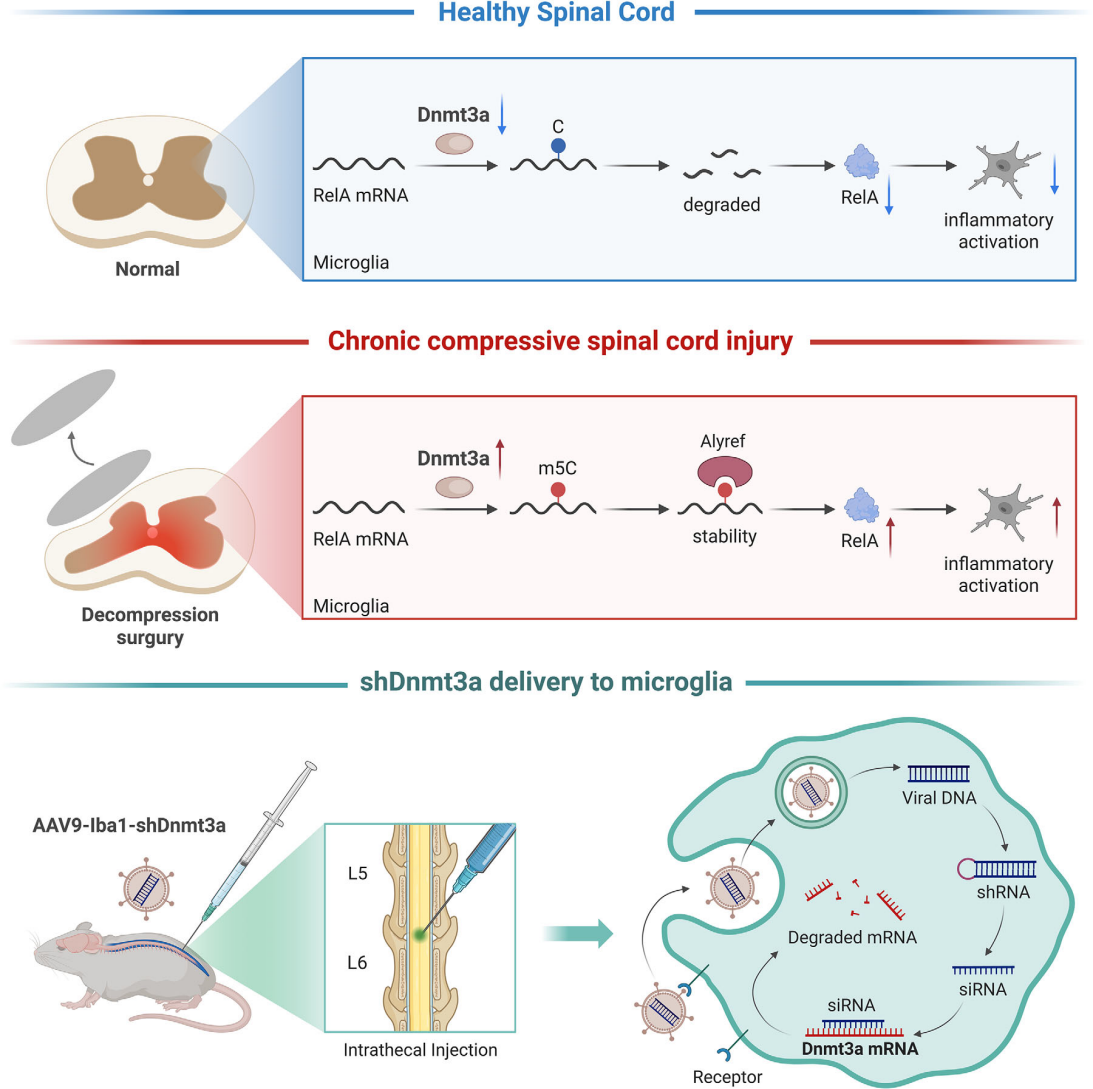
Proposed working model and therapeutic schematic for the Dnmt3a/RelA/m5C axis in microglia after decompression. (Top) Healthy spinal cord: microglial Dnmt3a is low; RelA mRNA carries little m5C and is rapidly degraded, limiting RelA protein and restraining inflammatory activation. (Middle) cCSCI after decompression: microglial Dnmt3a is upregulated, promoting m5C modification on RelA mRNA, which enhances transcript stability, sustains RelA expression, and drives inflammatory activation. (Bottom) Microglia‐targeted intervention: during decompression, intrathecal delivery of AAV‐shDnmt3a at L5–L6 enables shRNA processing to siRNA in microglia, degrading Dnmt3a mRNA and thereby reducing RelA.

In conclusion, we identified a pro‐inflammatory microglial subcluster (C1) in the post‐decompression spinal cord that displayed the most prominent m5C‐regulatory signature among all of the microglial populations. Integrated transcriptomic and epitranscriptomic analyses revealed a Dnmt3a/m5C/RelA signaling axis, wherein Dnmt3a is associated with increased m5C enrichment on RelA transcripts and enhanced RelA mRNA stability, thereby sustaining NF‐κB activation and maintaining a pro‐inflammatory microglial state. Genetic suppression of microglial Dnmt3a attenuated neuroinflammation and improved neurological recovery following decompression in cCSCI (Figure 8). Collectively, this study bridges a critical knowledge gap between surgical decompression and incomplete functional recovery by linking microglial subpopulation dynamics to m5C‐mediated stabilization of pro‐inflammatory transcripts and suggesting that the microglial Dnmt3a/m5C/RelA axis may represent a promising therapeutic target that warrants further translational investigation.

## Author Contributions

T. Qin contributed to writing – original draft, writing – review and editing, methodology, investigation, data curation, formal analysis, and conceptualization. Y. Jiang and Y. Xie contributed to writing – review and editing, methodology, investigation, and conceptualization. H. Qu contributed to writing – review and editing, data curation, formal analysis, and methodology. W. Yan, J. Chen, and Y. Chen contributed to validation, data curation, and formal analysis. H. Li contributed to formal analysis and visualization. N. Feng contributed to writing – review and editing, and funding acquisition. J. Wu contributed to writing – review and editing, and visualization. C. Zhang, Z. Huang, and M. Shi contributed to investigation and data curation. Z. Deng contributed to resources and methodology. G. Xiao contributed to writing – review and editing, supervision, project administration, methodology, and conceptualization. H. Long contributed to writing – review and editing, supervision, project administration, methodology, funding acquisition, and conceptualization.

## Funding

This study was funded by the National Natural Science Foundation of China (82302031), the Introduction of Talent Research Initiation Fund of Shenzhen People's Hospital (20200322‐1, Houqing Long), Guangdong Provincial Clinical Research Center for Geriatrics (2023B1111010012), Shenzhen Clinical Research Center for Geriatrics (LCYSSQ20210621092537007), and the National Key Clinical Specialty Construction Project (Department of Geriatrics).

## Ethics Declarations

The animal study was approved by the Institutional Animal Care and Use Committee of Shenzhen People’ s Hospital (AUP251030LHQ071601). For human study: not applicable.

## Conflicts of Interest

The authors declare that they do not have any conflicts of interest.

## Supporting information




**Supporting File**: advs75995‐sup‐0001‐SuppMat.docx.

## Data Availability

The data that support the findings of this study will be made available by the corresponding authors upon reasonable request.

## References

[1] R. Bartels , “A New Dimension in Degenerative Cervical Myelopathy,” The Lancet Neurology 20, no. 2 (2021): 82–83. 33357511 10.1016/S1474-4422(20)30454-3

[2] J. J. Wardropper , A. K. Demetriades , and D. B. Anderson , “Raising Awareness of Degenerative Cervical Myelopathy,” The Lancet Neurology 24, no. 4 (2025): 286–287. 40120607 10.1016/S1474-4422(25)00074-2

[3] B. M. Davies , O. D. Mowforth , E. K. Smith , and M. R. Kotter , “Degenerative Cervical Myelopathy,” Bmj 360 (2018): k186. 29472200 10.1136/bmj.k186 PMC6074604

[4] C. S. Ahuja , J. R. Wilson , S. Nori , et al., “Traumatic Spinal Cord Injury,” Nature Reviews Disease Primers 3 (2017): 17018. 10.1038/nrdp.2017.18 28447605

[5] J. H. Badhiwala , C. S. Ahuja , M. A. Akbar , et al., “Degenerative Cervical Myelopathy — Update and Future Directions,” Nature Reviews Neurology 16, no. 2 (2020): 108–124. 31974455 10.1038/s41582-019-0303-0

[6] B. Xu , H. Yin , and M. Feng , “Toward A Personalized Approach: The Promising Horizon Of Degenerative Cervical Myelopathy,” International Journal of Surgery 110, no. 8 (2024): 5276–5277. 38716921 10.1097/JS9.0000000000001569 PMC11325900

[7] A. Desimone , J. Hong , S. T. Brockie , W. Yu , A. M. Laliberte , and M. G. Fehlings , “The Influence of ApoE4 on the Clinical Outcomes and Pathophysiology of Degenerative Cervical Myelopathy,” JCI Insight 6, no. 15 (2021): 149227. 10.1172/jci.insight.149227 PMC8410082 34369386

[8] B. N. R. Jaja , C. D. Witiw , E. M. Harrington , et al., “Analysis of Recovery Trajectories in Degenerative Cervical Myelopathy to Facilitate Improved Patient Counseling and Individualized Treatment Recommendations,” Journal of Neurosurgery Spine 17 (2023): 1–9. 10.3171/2023.1.SPINE221053 36933253

[9] H. W. Kim , H. Yong , and G. K. H. Shea , “Blood‐spinal Cord Barrier Disruption in Degenerative Cervical Myelopathy,” Fluids and Barriers of the CNS 20, no. 1 (2023): 68. 37743487 10.1186/s12987-023-00463-y PMC10519090

[10] T. Hirai , K. Uchida , H. Nakajima , et al., “The Prevalence and Phenotype of Activated Microglia/Macrophages Within the Spinal Cord of the Hyperostotic Mouse (twy/twy) Changes in Response to Chronic Progressive Spinal Cord Compression: Implications for human Cervical Compressive Myelopathy,” PLoS ONE 8, no. 5 (2013): 64528. 10.1371/journal.pone.0064528 PMC3663759 23717624

[11] M. Takano , S. Kawabata , Y. Komaki , et al., “Inflammatory Cascades Mediate Synapse Elimination in Spinal Cord Compression,” Journal of Neuroinflammation 11 (2014): 40. 24589419 10.1186/1742-2094-11-40 PMC3975877

[12] N. J. Wrobel , Q. Shen , D. H. Kim , et al., “Long‐term Dynamics of the Spinal Cord Injury Neuroinflammatory Response and Sensory Dysfunction in Female Mice,” Brain, Behavior, and Immunity 129 (2025): 143–156. 40441542 10.1016/j.bbi.2025.05.024

[13] R. Wang , L. Ding , Y. Lin , et al., “The Quiet Giant: Identification, Effectors, Molecular Mechanism, Physiological and Pathological Function in mRNA 5‐methylcytosine Modification,” International Journal of Biological Sciences 20, no. 15 (2024): 6241–6254. 39664561 10.7150/ijbs.101337 PMC11628344

[14] H. Song , J. Zhang , B. Liu , et al., “Biological Roles of RNA m5C Modification and Its Implications in Cancer Immunotherapy,” Biomarker Research 10, no. 1 (2022): 15. 35365216 10.1186/s40364-022-00362-8 PMC8973801

[15] L. Zhang , Y. Li , L. Li , et al., “Detection, Molecular Function and Mechanisms of m5C in Cancer,” Clinical and Translational Medicine 15, no. 3 (2025): 70239. 10.1002/ctm2.70239 PMC11862898 40008496

[16] R. Li , H. Chen , C. Li , et al., “The Prognostic Value and Immune Landscaps of m6A/m5C‐Related lncRNAs Signature in the Low Grade Glioma,” BMC Bioinformatics [Electronic Resource] 24, no. 1 (2023): 274. 37403043 10.1186/s12859-023-05386-x PMC10320943

[17] Z. Xiao , Z. Wang , H. Jiang , Y. Liu , and W. Guo , “Tetramethylpyrazine Promotes Functional Recovery After Spinal Cord Injury by Regulating 5‐Methylcytosine RNA Modification via the NSUN2‐XBP1 Axis,” Molecular Neurobiology 63, no. 1 (2025): 74. 41254347 10.1007/s12035-025-05520-1

[18] L. Cao , W. J. Pi , Q. Zhang , and Q. Li , “Molecular Characterization and Clinical Characteristics of m5C‐Based RNA Methylation in Spinal Cord Injury: Validated by qPCR,” International Journal of Genomics 2022 (2022): 1–26. 10.1155/2022/5433860 PMC9794433 36582430

[19] Y. Xie , Y. Peng , T. Qin , et al., “AGAPIR: A Novel PIWI‐Interacting RNA Enhancing Post‐Decompression Angiogenesis in Degenerative Cervical Myelopathy,” Advancement of Science 12 (2025): 04246. 10.1002/advs.202504246 PMC12622476 40820968

[20] D. M. Basso , L. C. Fisher , A. J. Anderson , L. B. Jakeman , D. M. McTigue , and P. G. Popovich , “Basso Mouse Scale for Locomotion Detects Differences in Recovery After Spinal Cord Injury in Five Common Mouse Strains,” Journal of Neurotrauma 23, no. 5 (2006): 635–659. 16689667 10.1089/neu.2006.23.635

[21] I. Korsunsky , N. Millard , J. Fan , et al., “Fast, Sensitive and Accurate Integration of Single‐cell Data With Harmony,” Nature Methods 16, no. 12 (2019): 1289–1296. 31740819 10.1038/s41592-019-0619-0 PMC6884693

[22] G. Yu , L. G. Wang , Y. Han , and Q. Y. He , “clusterProfiler: an R Package for Comparing Biological Themes Among Gene Clusters,” OMICS: A Journal of Integrative Biology 16, no. 5 (2012): 284–287. 22455463 10.1089/omi.2011.0118 PMC3339379

[23] X. Qiu , Q. Mao , Y. Tang , et al., “Reversed Graph Embedding Resolves Complex Single‐cell Trajectories,” Nature Methods 14, no. 10 (2017): 979–982. 28825705 10.1038/nmeth.4402 PMC5764547

[24] M. Mizrachi and B. Diamond , “Impact of Microglia Isolation and Culture Methodology on Transcriptional Profile and Function,” Journal of Neuroinflammation 21, no. 1 (2024): 87. 38589917 10.1186/s12974-024-03076-w PMC11000335

[25] C. I. Gutiérrez‐Román and O. Medina‐Contreras , “Rapid and Efficient Enrichment of Mouse Spinal Cord Microglia,” Journal of visualized experiments: JoVE 22, no. 199 (2023): 65961. 10.3791/65961 37811962

[26] J. Wang , X. Deng , T. Jian , et al., “DNA Methyltransferase 1 Modulates Mitochondrial Function Through Bridging m5C RNA Methylation,” Molecular Cell 85, no. 10 (2025): 1999–2016.e11. 40328247 10.1016/j.molcel.2025.04.019

[27] S. M. Karim , D. W. Cadotte , J. R. Wilson , et al., “Effectiveness of Surgical Decompression in Patients With Degenerative Cervical Myelopathy: Results of the Canadian Prospective Multicenter Study,” Neurosurgery 89, no. 5 (2021): 844–851. 34382661 10.1093/neuros/nyab295

[28] L. Tetreault , B. Kopjar , P. Côté , P. Arnold , and M. G. Fehlings , “A Clinical Prediction Rule for Functional Outcomes in Patients Undergoing Surgery for Degenerative Cervical Myelopathy,” The Journal of Bone and Joint Surgery‐American Volume 97, no. 24 (2015): 2038–2046. 26677238 10.2106/JBJS.O.00189

[29] H. L. Harkey , O. al‐Mefty , I. Marawi , D. F. Peeler , D. E. Haines , and L. F. Alexander , “Experimental Chronic Compressive Cervical Myelopathy: Effects of Decompression,” Journal of Neurosurgery 83, no. 2 (1995): 336–341. 7616281 10.3171/jns.1995.83.2.0336

[30] T. P. Schmidt , K. Jütten , U. Bertram , et al., “Blood Spinal Cord Barrier Disruption Recovers In Patients With Degenerative Cervical Myelopathy After Surgical Decompression: A Prospective Cohort Study,” Scientific Reports 13, no. 1 (2023): 7389. 37149638 10.1038/s41598-023-34004-2 PMC10164176

[31] Z.‐Y. Li , Y.‐X. Dai , Z.‐M. Wu , et al., “Network Pharmacology Analysis and Animal Experiment Validation of Neuroinflammation Inhibition by Total Ginsenoside in Treating CSM,” Phytomedicine 126 (2024): 155073. 38417244 10.1016/j.phymed.2023.155073

[32] A. M. Laliberte , S. K. Karadimas , P. M. Vidal , K. Satkunendrarajah , and M. G. Fehlings , “Mir21 Modulates Inflammation and Sensorimotor Deficits in Cervical Myelopathy: Data From Humans and Animal Models,” Brain Communications 3, no. 1 (2021): fcaa234. 33604572 10.1093/braincomms/fcaa234 PMC7878254

[33] W. Song , R. Fu , Z. Yuan , et al., “Targeting RelA/NLRP3/CCL3 Axis Mitigates Microglia Inflammatory Response and Promotes Recovery After Spinal Cord Injury,” Brain, Behavior, and Immunity 129 (2025): 801–817. 40691997 10.1016/j.bbi.2025.07.015

[34] Y. Lin , M. Liu , P. Deng , and J. Zhang , “TET1 mediated m5C Modification of RelB Aggravates Cerebral Ischemia/Reperfusion‐induced Neuroinflammation Through Regulating Microglia Polarization,” Cellular Signalling 120 (2024): 111210. 38705503 10.1016/j.cellsig.2024.111210

[35] M. Cheray , A. Etcheverry , C. Jacques , et al., “Cytosine Methylation of Mature microRNAs Inhibits Their Functions and Is Associated With Poor Prognosis in Glioblastoma Multiforme,” Molecular Cancer 19, no. 1 (2020): 36. 32098627 10.1186/s12943-020-01155-z PMC7041276

[36] H. Chen , H. Yang , X. Zhu , et al., “m^5^C modification of mRNA Serves a DNA Damage Code to Promote Homologous Recombination,” Nature Communications 11, no. 1 (2020): 2834. 10.1038/s41467-020-16722-7 PMC7275041 32503981

[37] H. Yang , E. M. Lachtara , X. Ran , et al., “The RNA m^5^C Modification in R‐loops as an off Switch of Alt‐NHEJ,” Nature Communications 14, no. 1 (2023): 6114. 10.1038/s41467-023-41790-w PMC10542358 37777505

[38] Q. Dai , C. Ye , I. Irkliyenko , et al., “Ultrafast Bisulfite Sequencing Detection of 5‐methylcytosine in DNA and RNA,” Nature Biotechnology 42, no. 10 (2024): 1559–1570. 10.1038/s41587-023-02034-w PMC11217147 38168991

[39] L. Fang , W. Wang , G. Li , et al., “CIGAR‐seq, a CRISPR/Cas‐based method for unbiased screening of novel mRNA modification regulators,” Molecular Systems Biology 16, no. 11 (2020): 10025. 10.15252/msb.202010025 PMC7701898 33251765

[40] H. Mao , X. Zhao , and S. C. Sun , “NF‐κB in Inflammation and Cancer,” Cellular & Molecular Immunology 22, no. 8 (2025): 811–839. 40562870 10.1038/s41423-025-01310-w PMC12310982

[41] T. Ganbold , Q. Bao , J. Zandan , A. Hasi , and H. Baigude , “Modulation of Microglia Polarization Through Silencing of NF‐κB p65 by Functionalized Curdlan Nanoparticle‐Mediated RNAi,” ACS Applied Materials & Interfaces 12, no. 10 (2020): 11363–11374. 32073249 10.1021/acsami.9b23004

[42] Z. Lin , C. Chen , D. Yang , J. Ding , G. Wang , and H. Ren , “DJ‐1 Inhibits Microglial Activation and Protects Dopaminergic Neurons In vitro and In vivo Through Interacting With Microglial p65,” Cell Death & Disease 12, no. 8 (2021): 715. 34274951 10.1038/s41419-021-04002-1 PMC8286256

[43] Y. Shao , Y. Chen , X. Lan , et al., “Morin Regulates M1/M2 Microglial Polarization via NF‐κB p65 to Alleviate Vincristine‐Induced Neuropathic Pain,” Drug Design, Development and Therapy 18 (2024): 3143–3156. 39071815 10.2147/DDDT.S459757 PMC11278053

[44] X. X. Wang , B. Zhang , R. Xia , and Q. Y. Jia , “Inflammation, Apoptosis and Autophagy as Critical Players in Vascular Dementia,” European Review for Medical and Pharmacological Sciences 24, no. 18 (2020): 9601–9614. 33015803 10.26355/eurrev_202009_23048

[45] F. Li , J. Li , P.‐H. Wang , et al., “SARS‐CoV‐2 Spike Promotes Inflammation and Apoptosis Through Autophagy by ROS‐suppressed PI3K/AKT/mTOR Signaling,” Biochimica et Biophysica Acta (BBA)—Molecular Basis of Disease 1867, no. 12 (2021): 166260. 34461258 10.1016/j.bbadis.2021.166260 PMC8390448

[46] M. G. Fehlings , N. Evaniew , P. V. Ter Wengel , et al., “AO Spine Clinical Practice Recommendations for Diagnosis and Management of Degenerative Cervical Myelopathy: Evidence Based Decision Making—A Review of Cutting Edge Recent Literature Related to Degenerative Cervical Myelopathy,” Global Spine Journal 15, no. 5 (2025): 2585–2593. 40257837 10.1177/21925682251331050 PMC12012498

[47] J. J. Levett , M. Georgiopoulos , S. Martel , et al., “Pharmacological Treatment of Degenerative Cervical Myelopathy: A Critical Review of Current Evidence,” Neurospine 21, no. 2 (2024): 375–400. 38955515 10.14245/ns.2448140.070 PMC11224758

[48] J. R. Mendell , S. A. Al‐Zaidy , L. R. Rodino‐Klapac , et al., “Current Clinical Applications of In vivo Gene Therapy With AAVs,” Molecular Therapy 29, no. 2 (2021): 464–488. 33309881 10.1016/j.ymthe.2020.12.007 PMC7854298

[49] L. Kang , S. Jin , J. Wang , et al., “AAV Vectors Applied to the Treatment of CNS Disorders: Clinical Status and Challenges,” Journal of Controlled Release 355 (2023): 458–473. 36736907 10.1016/j.jconrel.2023.01.067

[50] D. X. Bharucha‐Goebel , J. J. Todd , D. Saade , et al., “Intrathecal Gene Therapy for Giant Axonal Neuropathy,” New England Journal of Medicine 390, no. 12 (2024): 1092–1104. 38507752 10.1056/NEJMoa2307952 PMC11973737

