# Supplementary material for: Targeting Dnmt3a/m5C/RelA Axis Attenuates Microglia Inflammatory Response and Improves Postoperative Recovery in Chronic Compressive Cervical Spinal Cord Injury
Source: Adv Sci (Weinh). 2026 Jun 9;13(48):e75995. doi: 10.1002/advs.75995 (PMC13336428; doi:10.1002/advs.75995)
Supplement: Supplementary file 1 [file ADVS-13-e75995-s001.docx]

**Supporting Information**

**Targeting Dnmt3a/m5C/RelA axis attenuates microglia inflammatory response and improves postoperative recovery in chronic compressive cervical spinal cord injury**

Tianyu Qin^1,2†^, Yuan Jiang^1,2,3†^, Yongheng Xie^1,2†^, Huanwei Qu^4†^, Wenbin Yan^1,2^, Jingle Chen^1,2^, Yining Chen^1,2^, Huayi Li^1,2^, Naibo Feng^1,2,5^, Jiajun Wu^6^, Chao Zhang^6^, Zhengqi Huang^6^, Ming Shi^6^, Zhihuai Deng^6^, Guozhi Xiao^7^*, Houqing Long^1,2,8^*

^1^ Division of Spine, Department of Orthopedic Surgery, Shenzhen People's Hospital (The Second Clinical Medical College, Jinan University, The First Affiliated Hospital, Southern University of Science and Technology), Shenzhen 518020, China.

^2^ Shenzhen Key Laboratory of Musculoskeletal Tissue Reconstruction and Function Restoration, Shenzhen 518020, China.

^3^ Division of Spine, Department of Orthopedic Surgery, Changde Hospital, Xiangya School of Medicine, Central South University, Changde 415000, China.

^4^ Department of Orthopedics, Tianjin Hospital, Tianjin University, Tianjin 300211, China.

^5^ Department of Trauma Orthopedics, Weifang People's Hospital, Shandong Second Medical University, Weifang 261000, China.

^6^ Department of Orthopedics, Sun Yat-sen Memorial Hospital, Sun Yat-sen University, Guangzhou 510120, China.

^7^ Department of Biochemistry, Homeostatic Medicine Institute, School of Medicine, Guangdong Provincial Key Laboratory of Cell Microenvironment and Disease Research, Southern University of Science and Technology, Shenzhen 518055, China.

^8^ Guangdong Provincial Clinical Research Center for Geriatrics, Shenzhen Clinical Research Center for Geriatrics, Department of Geriatrics, Shenzhen People's Hospital, Shenzhen 518020, China.

^†^These authors contributed equally to this work.

*****Corresponding authors:

[longhouqing@szhospital.com](mailto:longhouqing@szhospital.com);

[xiaogz@sustech.edu.cn](mailto:xiaogz@sustech.edu.cn)

# 1. **Supplementary figures**

**Figure S1**. (A) Representative flow cytometry plots showing time-dependent increases in CD86⁺ microglial populations after stimulation with LPS for 6 h, 12 h, and 24 h. CTR, untreated control. (B) Quantitative analysis of CD86⁺ microglia proportions at each time point. Data are presented as means ± SDs; * *p* < 0.05, ** *p* < 0.01, *** *p* < 0.001, **** *p* < 0.0001.

**Figure S2**. (A) Dot-plot displaying the expression patterns of canonical marker genes across eight identified spinal-cord cell types. (B) Bar graph summarizing total cell counts of each major cell type derived from single-nucleus RNA-seq across all experimental groups. (C) Stacked bar chart showing the relative proportions of each cell type in the Sham, Compression (Comp), and Decompression (Decom) groups, revealing a marked expansion of microglia in the Comp group that partially subsided after decompression. (D) UMAP plot of six microglial subclusters (C0–C5) from Sham, Comp, and Decom spinal cords. (E) Stacked bar chart illustrating the relative proportions of six microglial subclusters (C0–C5) among groups, showing predominant enrichment of the C1 subcluster in both Comp and Decom conditions.

**Figure S3**. (A) Pseudotime trajectory of microglial differentiation constructed using Monocle. (B) Distribution of cells from Sham, Compression (Comp), and Decompression (Decom) groups along pseudotime. (C–I) Pie charts showing the proportions of cells from each experimental group within the seven pseudotime states. States 4–6 were markedly enriched in the Comp and Decom groups, suggesting activation of disease-related microglial programs. (J) Pseudotime distribution of the six microglial subclusters (C0–C5). (K–Q) Pie charts depicting the proportions of microglial subclusters (C0–C5) across the seven pseudotime states. The C1 subcluster predominated in States 4–6, corresponding to inflammatory activation phases. (R) KEGG enrichment analysis of C1 state-associated genes. (S) Hallmark pathway enrichment of C1-upregulated genes.

**Figure S4**. (A–G) Pseudotime trajectory plots showing m⁵C enrichment scores of major spinal cord cell types—including astrocytes (A), endothelial cells (B), fibroblasts (C), neurons (D), oligodendrocytes (E), oligodendrocyte precursor cells (OPCs; F), and pericytes (G)—across the Sham, Compression (Comp), and Decompression (Decom) groups.

**Figure S5**. (A–P) Boxplots showing expression levels of representative m5C methyltransferases, demethylases, and binding proteins among six microglial subclusters (C0–C5). The analyzed genes include Nsun6 (A), Tet1 (B), Tet3 (C), Alkbh1 (D), Nop2 (E), Nsun2 (F), Nsun3 (G), Nsun4 (H), Nsun5 (I), Nsun7 (J), Dnmt1 (K), Trdmt1 (L), Dnmt3b (M), Alyref (N), Tet2 (O), and Ybx1 (P).

**Figure S6**. (A–P) Boxplots showing the expression levels of m5C regulatory genes in microglia from Sham, Compression (Comp), and Decompression (Decom) groups, including Nsun4 (A), Nsun5 (B), Nsun7 (C), Tet1 (D), Dnmt3b (E), Nop2 (F), Nsun2 (G), Nsun3 (H), Alkbh1 (I), Nsun6 (J), Alyref (K), Dnmt1 (L), Tet2 (M), Tet3 (N), Trdmt1 (O), and Ybx1 (P). (Q) Heatmap illustrating the expression correlation between Dnmt3a and its positively associated genes (Lilrb4a, Lgals3, Rab7b, Cd9, Csf1, Abca1, Hif1a) across microglial subclusters (C0–C5). The C1 subcluster exhibited the highest co-expression intensity (red), supporting the strong association between Dnmt3a and immune activation–related pathways in microglia during cCSCI progression.

**Figure S7**. (A, B) Western blot analysis and quantification of Dnmt3a protein expression in primary microglia transfected with three independent siRNAs targeting Dnmt3a (siDnmt3a-1, siDnmt3a-2, and siDnmt3a-3). (C) Western blot analysis confirming the silencing efficiency of Dnmt3a in primary microglia transfected with siDnmt3a-2 compared to negative control (siNC) and untreated control (CTR). Actin was used as a loading control. (D) Quantification of Dnmt3a protein expression normalized to actin showing that siDnmt3a-2 significantly reduced Dnmt3a expression compared with siNC, confirming effective gene silencing. Data are presented as means ± SDs. **** *p* < 0.0001; ns, not significant.

**Figure S8**. (A) Scatter plot showing differentially expressed genes (DEGs) between siDnmt3a and siNC groups. Red and blue dots represent upregulated and downregulated genes, respectively, while gray dots indicate genes with no significant change. (B) GO enrichment analysis of downregulated DEGs, categorized into BP, CC, and MF terms. Enriched terms include regulation of inflammatory response, positive regulation of cytokine production, cytokine receptor binding, and immune receptor activity, suggesting suppressed pro-inflammatory signaling following Dnmt3a knockdown. (C) KEGG enrichment analysis of upregulated DEGs, highlighting pathways associated with arginine and proline metabolism, peroxisome, and FoxO signaling, implying metabolic and stress response adaptations after Dnmt3a silencing. (D) GO enrichment analysis of upregulated DEGs showing activation of biological processes related to oxidative metabolism, nucleotide biosynthesis, and myeloid cell differentiation.

**Figure S9**. KEGG pathway enrichment map of the NF-κB signaling pathway based on RNA-seq analysis comparing siDnmt3a versus siNC groups. Genes significantly downregulated by Dnmt3a silencing are highlighted in green, with color intensity representing the magnitude of log2 fold change. Downregulated targets include key inflammatory mediators (IL-1β, TNFα, COX2, RelA, Nfkb1, Nfkb2, MIP-1β, and VCAM1), indicating that Dnmt3a knockdown markedly suppresses canonical NF-κB pathway activation and downstream inflammatory gene expression.

**Figure S10**. (A) RIP-qPCR analysis showing enrichment of RelA mRNA in anti-Dnmt3a, anti-NSUN2, and anti-NSUN6 immunoprecipitates relative to IgG controls. (B, C) Western blot analysis and quantification of YBX1 protein expression in primary microglia transfected with three independent siRNAs targeting YBX1 (siYBX1-1, siYBX1-2, and siYBX1-3). (D, E) Western blot analysis and quantification of Alyref protein expression in primary microglia transfected with three independent siRNAs targeting Alyref (siAlyref-1, siAlyref-2, and siAlyref-3). (F) Actinomycin D chase assay showing that YBX1 knockdown did not significantly alter the decay rate or half-life of RelA mRNA compared with siNC-transfected cells. (G, H) Western blot analysis and quantification showing that YBX1 silencing did not markedly affect RelA protein expression. (I) Actinomycin D chase assay showing that Alyref knockdown accelerated RelA mRNA degradation and shortened its half-life compared with siNC-transfected cells. Symbols indicate comparisons: * vs siAlyref-1; # vs siAlyref-1. (J, K) Western blot analysis and quantification showing that Alyref silencing reduced RelA protein abundance. (L) RIP-qPCR analysis showing significant enrichment of RelA mRNA in anti-Alyref immunoprecipitates relative to the IgG control. Data are presented as means ± SDs. * *p* < 0.05, ** *p* < 0.01, *** *p* < 0.001, **** *p* < 0.0001, ns = not significant.

**Figure S11**. (A) Western blot analysis confirming RelA overexpression efficiency in primary microglia transfected with the oe-RelA plasmid compared to control (CTR) and empty vector (oe-NC) groups. Actin served as the loading control. (B) Densitometric quantification showing that RelA protein expression was significantly increased (~4.5-fold) in the oe-RelA group relative to CTR and oe-NC groups, confirming successful transfection and protein overexpression. Data are presented as means ± SDs. **** *p* < 0.0001, ns = not significant.

**Figure S12**. (A–C) Behavioral tests including BMS score, inclined-plane test, and forelimb wire-hanging test performed within eight weeks after intrathecal injection of AAV-shDnmt3a or AAV-shNC. (D) Body-weight monitoring during the eight-week experimental period, showing steady growth without intergroup differences. (E) Representative immunofluorescence images of spinal-cord sections showing colocalization of Dnmt3a (red) with the microglial marker Iba1 (green). Scale bar = 30 μm. (F) Quantification of Dnmt3a⁺ Iba1⁺ microglia, showing that AAV-shDnmt3a significantly decreased Dnmt3a expression compared with Decom and AAV-shNC groups. (G-L) Representative immunofluorescence images showing Dnmt3a co-staining with NeuN, GFAP, or Olig2 in spinal cord sections from the sham, Decom, Decom + AAV-shNC, and Decom + AAV-shDnmt3a groups. Scale bar = 30 μm. Data are presented as means ± SDs. **** *p* < 0.0001.

# 2. **Supplementary table**

| **Table S1:** m5C regulator | | |
| --- | --- | --- |
| Writer | Reader | Eraser |
| Nsun1 (Nop2) | Alyref | Tet1 |
| Nsun2 | Ybx1 | Tet2 |
| Nsun3 |  | Tet3 |
| Nsun4 |  | Alkbh1 |
| Nsun5 |  |  |
| Nsun6 |  |  |
| Nsun7 |  |  |
| Dnmt1 |  |  |
| Dnmt2 (Trdmt1) |  |  |
| Dnmt3a |  |  |
| Dnmt3b |  |  |

| **Table S2:** Gene–Pathway Associations | | | | |
| --- | --- | --- | --- | --- |
| feature1 | feature2 | corr | pvalue | type |
| Lilrb4a | Dnmt3a | 0.145600832 | 3.84064E-62 | GOBP_Activation_of_immune_response |
| Lilrb4a | Dnmt3a | 0.145600832 | 3.84064E-62 | GOBP_Immune_response_activating_signaling_pathway |
| Cd9 | Dnmt3a | 0.144627292 | 2.49344E-61 | IL6_JAK_STAT3_SIGNALING |
| Lgals3 | Dnmt3a | 0.128785455 | 6.71413E-49 | GOBP_Activation_of_immune_response |
| Lgals3 | Dnmt3a | 0.128785455 | 6.71413E-49 | GOBP_Immune_response_activating_signaling_pathway |
| Csf1 | Dnmt3a | 0.127382592 | 7.18498E-48 | IL6_JAK_STAT3_SIGNALING |
| Csf1 | Dnmt3a | 0.127382592 | 7.18498E-48 | TNFA_SIGNALING_VIA_NFKB |
| Csf1 | Dnmt3a | 0.127382592 | 7.18498E-48 | INFLAMMATORY_RESPONSE |
| Hif1a | Dnmt3a | 0.121295096 | 1.54875E-43 | INFLAMMATORY_RESPONSE |
| Abca1 | Dnmt3a | 0.105884084 | 1.57251E-33 | TNFA_SIGNALING_VIA_NFKB |
| Abca1 | Dnmt3a | 0.105884084 | 1.57251E-33 | INFLAMMATORY_RESPONSE |
| Rab7b | Dnmt3a | 0.103269881 | 5.725E-32 | GOBP_Activation_of_immune_response |
| Rab7b | Dnmt3a | 0.103269881 | 5.725E-32 | GOBP_Immune_response_activating_signaling_pathway |

| **Table S3:** Gene expression in the NF-κB signaling pathway | | | | | |
| --- | --- | --- | --- | --- | --- |
| log2FoldChange | pvalue | siDnmt3a | siNC | gene_name | GeneID |
| -7.363181893 | 9.82E-109 | 6.845458138 | 1151.748159 | Cxcl2 | 20310 |
| -7.236347554 | 5.74E-85 | 5.3153133 | 815.4744575 | Il1b | 16176 |
| -5.851018709 | 5.76E-121 | 14.39444274 | 827.1681291 | Rela | 19697 |
| -5.740014621 | 3.66E-133 | 17.00050296 | 916.9394344 | Traf1 | 22029 |
| -3.985717332 | 0.030202932 | 0 | 2.79258961 | Cxcl3 | 330122 |
| -3.750802449 | 2.53E-122 | 57.10571545 | 767.9476425 | Ptgs2 | 19225 |
| -3.539412429 | 6.36E-231 | 295.7762905 | 3443.620233 | Tnf | 21926 |
| -3.323961658 | 9.69E-05 | 1.56922594 | 15.88103338 | Lta | 16992 |
| -2.903366888 | 0.043474595 | 0.683432608 | 4.876497171 | Bcl2a1a | 12044 |
| -2.851046561 | 1.43E-66 | 62.9452613 | 453.1717279 | Tnfaip3 | 21929 |
| -2.534774194 | 1.74E-101 | 251.7168361 | 1456.823451 | Nfkbia | 18035 |
| -2.476041614 | 5.16E-124 | 557.1144668 | 3098.584645 | Ccl4 | 20303 |
| -2.424888277 | 5.27E-45 | 89.8413891 | 484.6060502 | Bcl2a1d | 12047 |
| -2.060302187 | 3.83E-33 | 102.1527457 | 425.3721622 | Cd40 | 21939 |
| -1.866346497 | 4.88E-20 | 68.95458595 | 252.631184 | Bcl2a1b | 12045 |
| -1.671812093 | 0.011113469 | 4.98991523 | 16.0041773 | Ltb | 16994 |
| -1.559435505 | 4.10E-06 | 18.97555697 | 55.61375461 | Tnfsf14 | 50930 |
| -1.442420533 | 2.51E-57 | 1146.696615 | 3115.41631 | Nfkb1 | 18033 |
| -1.318362582 | 3.02E-39 | 544.2567108 | 1356.858672 | Icam1 | 15894 |
| -1.304135099 | 8.00E-15 | 142.3971617 | 351.42621 | Pias4 | 59004 |
| -1.296978774 | 1.86E-22 | 219.3933377 | 538.5849764 | Relb | 19698 |
| -1.265862644 | 3.05E-27 | 974.5705791 | 2342.41105 | Nfkb2 | 18034 |
| -1.217729247 | 1.56E-39 | 1429.161486 | 3323.442942 | Cd14 | 12475 |
| -1.211844041 | 1.52E-31 | 750.0762355 | 1736.939895 | Bcl2l1 | 12048 |
| -1.121427213 | 1.18E-20 | 360.4689653 | 783.7943698 | Blnk | 17060 |
| -1.036856021 | 3.32E-09 | 131.1155869 | 269.5551195 | Gadd45a | 13197 |

| **Table S4:** Primary antibodies Used in the Present Study | | | | | |
| --- | --- | --- | --- | --- | --- |
| Name | Origin | Applications | Lot Number | Company | RRID |
| Dnmt3a | Rabbit | WB / IF | ab188470 | Abcam | AB_3073896 |
| RelA | Rabbit | WB / IF | ab16502 | Abcam | AB_443394 |
| Iba1 | Mouse | IF | ab283319 | Abcam | AB_2924797 |
| iNOS | Rabbit | WB / IF | ab178945 | Abcam | AB_2861417 |
| COX2 | Rabbit | WB / IF | ab179800 | Abcam | AB_2894871 |
| CD86 | Rat | IF | ab119857 | Abcam | AB_10902800 |
| CD11b | Rat | Flow cytometry | 561689 | BD Biosciences | AB_10893803 |
| CD45 | Rat | Flow cytometry | 559864 | BD Biosciences | AB_398672 |
| CD86 | Rat | Flow cytometry | 558703 | BD Biosciences | AB_2075114 |
| Dnmt3a | Rabbit | RIP | ab307503 | Abcam | AB_3105875 |
| NSUN2 | Rabbit | RIP | ab259941 | Abcam | AB_3741454 |
| NSUN6 | Rabbit | RIP | ab307430 | Abcam | AB_3741453 |
| YBX1 | Rabbit | WB | ab255606 | Abcam | AB_3741455 |
| ALYREF | Rabbit | WB | ab202894 | Abcam | AB_3094572 |
| NEUN | Mouse | IF | 94403 | CST | AB_2904530 |
| GFAP | Mouse | IF | 3655 | CST | AB_2263284 |
| Olig2 | Mouse | IF | 39588 | CST | AB_3741539 |
| β-actin | Rabbit | WB | 20536-1-AP | Proteintech | AB_10700003 |
| m5C | Rabbit | Dot Blot / MeRIP | ab214727 | Abcam | AB_2802117 |

| **Table S5:** Sequence of siRNA Used in the Present Study | | |
| --- | --- | --- |
| Sequence | Sense (5'-3') | Anti-sense (5'-3') |
| siDnmt3a-2 | AGAAGAAGAGAAGAAUCCUTT | AGGAUUCUUCUCUUCUUCUTT |
| siYBX1-2 | GUCAAAUGGUUCAAUGUAATT | UUACAUUGAACCAUUUGACTT |
| siYBX1-3 | UGAUGUUGUUGAAGGAGAATT | UUCUCCUUCAACAACAUCATT |
| siAlyref-1 | GGAAGCUGCUGGUGUCCAATT | UUGGACACCAGCAGCUUCCTT |
| siAlyref-2 | GAAACAACUUCCCGACAAATT | UUUGUCGGGAAGUUGUUUCTT |
| siNC | UUCUCCGAACGUGUCACGUdTdT | ACGUGACACGUUCGGAGAAdTdT |

| **Table S6:** Primers Used in the Present Study | | |
| --- | --- | --- |
| Gene | Sense (5'-3') | Anti-sense (5'-3') |
| Dnmt3a | CACCAGCCAAGAAACCCAGA | CATTGAGGCTCCCACATGAGATA |
| RelA | CAGTGAGCCCATGGAGTTCC | GGGTTCAGTTGGTCCATTGAAA |
| RelB | CCGTGCACAGGTGGTGAG | GGTGCACATCAGCTTGAGAGAA |
| Nfkb2 | CCATGCTGTGCCCCAAAT | CTCTCTGGGGCCTCCACTTC |
| Nfkb1 | ACAAGGAGCAGGACATGGGA | GGCCAGCAACATCTTCACATC |
| TNFα | CCCTCACACTCAGATCATCTTCT | GCTACGACGTGGGCTACAG |
| IL-6 | TAGTCCTTCCTACCCCAATTTCC | TTGGTCCTTAGCCACTCCTTC |
| iNOS | CTCAGCAGCATCCATGCAAA | AAGGTGAGCTGAACGAGGAGG |
| COX2 | CTGCCCGACACCTTCAACAT | CCAGCAATCTGTCTGGTGAATG |
